# *TLR7* gain-of-function genetic variation causes human lupus

**DOI:** 10.1038/s41586-022-04642-z

**Published:** 2022-04-27

**Authors:** Grant J. Brown, Pablo F. Cañete, Hao Wang, Arti Medhavy, Josiah Bones, Jonathan A. Roco, Yuke He, Yuting Qin, Jean Cappello, Julia I. Ellyard, Katharine Bassett, Qian Shen, Gaetan Burgio, Yaoyuan Zhang, Cynthia Turnbull, Xiangpeng Meng, Phil Wu, Eun Cho, Lisa A. Miosge, T. Daniel Andrews, Matt A. Field, Denis Tvorogov, Angel F. Lopez, Jeffrey J. Babon, Cristina Aparicio López, África Gónzalez-Murillo, Daniel Clemente Garulo, Virginia Pascual, Tess Levy, Eric J. Mallack, Daniel G. Calame, Timothy Lotze, James R. Lupski, Huihua Ding, Tomalika R. Ullah, Giles D. Walters, Mark E. Koina, Matthew C. Cook, Nan Shen, Carmen de Lucas Collantes, Ben Corry, Michael P. Gantier, Vicki Athanasopoulos, Carola G. Vinuesa

**Affiliations:** 1 Centre for Personalised Immunology, Department of Immunology and Infectious Disease, John Curtin School of Medical Research, Australian National University, Canberra, Australian Capital Territory Australia; 2 Research School of Biology, Australian National University, Canberra, Australian Capital Territory Australia; 3 China Australia Centre for Personalised Immunology, Shanghai Renji Hospital, Shanghai Jiaotong University, Shanghai, China; 4 Centre for Tropical Bioinformatics and Molecular Biology, Australian Institute of Tropical Health and Medicine, James Cook University, Cairns, Queensland Australia; 5 Centre for Cancer Biology, SA Pathology and the University of South Australia, Adelaide, South Australia Australia; 6 Division of Structural Biology, Walter and Eliza Hall Institute of Medical Research, Parkville, Victoria Australia; 7 Sección de Nefrología, Hospital Infantil Universitario Niño Jesús, Madrid, Spain; 8 Unidad de Terapias Avanzadas, Oncología, Hospital Infantil Universitario Niño Jesús, Madrid, Spain; 9 Fundación de Investigación Biomédica, Hospital Infantil Universitario Niño Jesús, Madrid, Spain; 10 Unidad de Reumatología, Hospital del Niño Jesus, Madrid, Spain; 11 Department of Pediatrics, Drukier Institute for Children’s Health, Weill Cornell Medical College, New York, NY USA; 12 Seaver Autism Center for Research and Treatment, Icahn School of Medicine at Mount Sinai, New York, NY USA; 13 Department of Psychiatry, Icahn School of Medicine at Mount Sinai, New York, NY USA; 14 Division of Child Neurology, Weill Cornell Medical College, New York-Presbyterian Hospital, New York, NY USA; 15 Division of Pediatric Neurology and Developmental Neuroscience, Department of Pediatrics, Baylor College of Medicine, Houston, TX USA; 16 Texas Children’s Hospital, Houston, TX USA; 17 Department of Molecular and Human Genetics, Baylor College of Medicine, Houston, TX USA; 18 Human Genome Sequencing Center, Baylor College of Medicine, Houston, TX USA; 19 Department of Pediatrics, Baylor College of Medicine, Houston, TX USA; 20 Shanghai Institute of Rheumatology, Renji Hospital, School of Medicine, Shanghai, Jiao Tong University (SJTUSM), Shanghai, China; 21 Centre for Innate Immunity and Infectious Diseases, Hudson Institute of Medical Research, Clayton, Victoria Australia; 22 Department of Molecular and Translational Science, Monash University, Clayton, Victoria Australia; 23 Department of Renal Medicine, The Canberra Hospital, Canberra, Australian Capital Territory Australia; 24 Department of Anatomical Pathology, The Canberra Hospital, Canberra, Australian Capital Territory Australia; 25 Center for Autoimmune Genomics and Etiology, Cincinnati Children’s Hospital Medical Center, Cincinnati, OH USA; 26 Departamento de Pediatría. Facultad de Medicina, Universidad Autónoma de Madrid (UAM), Madrid, Spain; 27 Francis Crick Institute, London, UK

**Keywords:** Autoimmunity, Central tolerance

## Abstract

Although circumstantial evidence supports enhanced Toll-like receptor 7 (TLR7) signalling as a mechanism of human systemic autoimmune disease^1–7^, evidence of lupus-causing *TLR7* gene variants is lacking. Here we describe human systemic lupus erythematosus caused by a *TLR7* gain-of-function variant. TLR7 is a sensor of viral RNA^8^,^9^ and binds to guanosine^10^–^12^. We identified a de novo, previously undescribed missense *TLR7*^*Y264H*^ variant in a child with severe lupus and additional variants in other patients with lupus. The *TLR7*^*Y264H*^ variant selectively increased sensing of guanosine and 2',3'-cGMP^10–12^, and was sufficient to cause lupus when introduced into mice. We show that enhanced TLR7 signalling drives aberrant survival of B cell receptor (BCR)-activated B cells, and in a cell-intrinsic manner, accumulation of CD11c^+^ age-associated B cells and germinal centre B cells. Follicular and extrafollicular helper T cells were also increased but these phenotypes were cell-extrinsic. Deficiency of MyD88 (an adaptor protein downstream of TLR7) rescued autoimmunity, aberrant B cell survival, and all cellular and serological phenotypes. Despite prominent spontaneous germinal-centre formation in *Tlr7*^*Y264H*^ mice, autoimmunity was not ameliorated by germinal-centre deficiency, suggesting an extrafollicular origin of pathogenic B cells. We establish the importance of TLR7 and guanosine-containing self-ligands for human lupus pathogenesis, which paves the way for therapeutic TLR7 or MyD88 inhibition.

## Abstract (web summary)

The missense *TLR7*^*Y264H*^ gain-of-function genetic variation causes systemic lupus erythematosus in humans and mice.

## Main

Although systemic lupus erythematosus (SLE) is generally a polygenic autoimmune disease, the discovery of monogenic lupus cases and rare pathogenic variants has provided important insights into disease mechanisms, including important roles of complement, type I interferons and B cell survival^13–15^. There is accumulating evidence that patients with SLE display phenotypes that are consistent with increased TLR7 signalling associated with elevated IgD^−^CD27^−^ double-negative B cells and, more specifically, the CXCR5^−^CD11c^+^ subset (also known as DN2 B cells or age-associated B cells (ABCs)) in the peripheral blood^1^, and excessive accumulation of extrafollicular helper T cells^16^. Genome-wide association studies have identified common polymorphisms in or near *TLR7* that segregate with SLE^2–4^. In mice, increased TLR7 signalling due to the duplication of the *TLR7*-encoding *Yaa* locus or to transgenic *TLR7* expression exacerbates autoimmunity^5,6^ and deletion of *TLR7* prevents or ameliorates disease in other lupus models^7^. Despite this mounting link between TLR7 and the pathogenesis of lupus, no human SLE cases due to *TLR7* variants have been reported to date. There is also conflicting evidence as to how TLR7 overexpression causes autoimmunity, particularly, the relative roles of TLR7-driven spontaneous germinal centres (GCs) versus the role of TLR7-driven double-negative B cells; the latter have been proposed to originate extrafollicularly and be pathogenic in lupus^1^. Most mouse lupus models in which TLR7 has a role in pathogenicity display increased formation of GCs and T follicular helper (T_FH_) cells^5,6^ and it has been proposed that TLR7 drives GCs enriched in self-reactive B cells^17^. However, recent reports have demonstrated that lupus can develop independently of GCs in mouse models in which disease is dependent on MyD88 signalling^18,19^.

TLR7 and TLR8 selectively detect a subset of RNA sequences^20–22^. On the basis of recent knowledge of how TLR8 senses RNA degradation products to trigger downstream signalling^23,24^, it is thought that one ligand-recognition site in TLR7 binds to guanosine or 2′,3′-cyclophosphate guanosine monophosphate (cGMP), derived from GTP degradation^10^, which synergizes with uridine-rich short RNAs binding to a second site^11,12^. Here we describe the action of a de novo *TLR7* single-residue gain-of-function (GOF) variant that increases the affinity of TLR7 for guanosine and cGMP, causing enhanced TLR7 activation and childhood-onset SLE.

## *TLR7* variants in patients with SLE

**Fig. 1:**
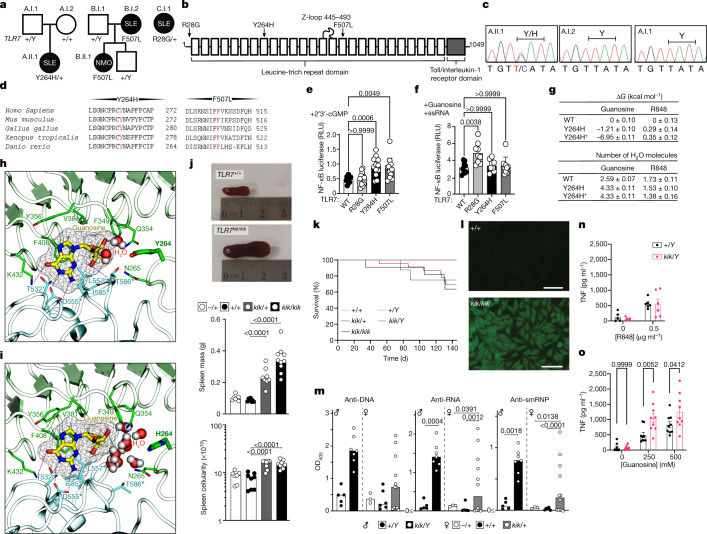
TLR7 variants in patients and mice with systemic autoimmunity and ligand-binding modelling. **a**, **b**, *TLR7* variants in families (**a**) and within protein domains^25^ (**b**). **c**, Sanger sequencing results. **d**, Conservation. **e**, **f**, NF-κB activity (ratio of NF-κB firefly to *Renilla* luciferase relative light units (RLU)) after human *TLR7* plasmid transfection into RAW264.7 cells treated with 2′,3′-cGMP (**e**) or guanosine plus ssRNA (**f**). Data are mean and s.d. *n* = 12 biological replicates or transfections shown as individual dots. Statistical significance was calculated using one-way analysis of variance (ANOVA) with Bonferroni correction for multiple comparisons, compared with wild-type *TLR7*. **g**, Binding free energy (Δ*G*; top) of guanosine and R848 relative to wild-type (WT) TLR7 for the Y264H and Y264H^+^ mutants, and the number (bottom) of bound water molecules within 3.5 Å of ligand tail hydrogen and oxygen atoms. Data are mean and s.e.m. **h**, **i**, Molecular dynamics simulations showing the binding pose of guanosine to wild-type (**h**) and Y264H (**i**) TLR7. Guanosine is shown in thick liquorice with yellow carbon atoms and mesh surface. TLR7 is shown as a ribbon representation with the guanosine-interacting residues shown as sticks. Individual protomers are coloured green and blue. The dashed lines indicate hydrogen bonds. Asterisks indicate residues belonging to the other protomer. Water molecules within 5 Å of both guanosine and residue 264 are shown. **j**, Spleens from *Tlr7*^*+/+*^ and *Tlr7*^*kik/kik*^ female mice (top). Bottom, median spleen mass and cellularity. **k**, The survival of kika mice. *n* = 12 (*Tlr7*^*+/+*^), *n* = 25 (*Tlr7*^*+/kik*^), *n* = 11 (*Tlr7*^*kik/kik*^),* n* = 11 (*Tlr7*^*+/Y*^),* n* = 7 (*Tlr7*^*kik/Y*^).** l**, Hep-2 immunofluorescence showing ANAs in 12-week-old kika mice. Representative of *n* > 10 mice. Scale bars, 100 µm. **m**, Serum antibodies to ssDNA (ANA), ssRNA and smRNP from kika mice (aged 12 weeks). OD_405_, optical density at 405 nm. Bars indicate median values. **n**, **o**, TNF production from mouse BMDMs treated with R848 (**n**) or guanosine (**o**). Data are mean ± s.e.m. The results represent *n* = 4 (**j**), *n* = 3 (**e**, **k**, **m**–**o**) or *n* = 2 (**f**) experiments. Statistical analysis was performed using Tukey multiple-comparison tests (**j**, **m**); and two-way ANOVA with Sidak (**o**). Exact *P* values are shown.

**Extended Data Fig. 1:**
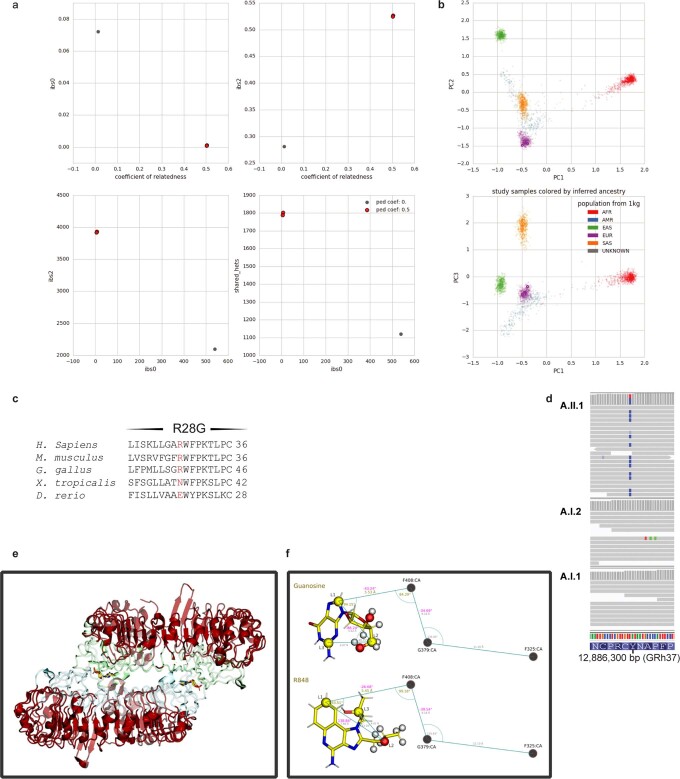
Confirmation of paternity in trio of proband with *TLR7* de novo variant and methods for molecular modelling. (**a**) Peddy diagrams used to establish relatedness. Each red dot represents a child/parent pair (child mother and child father). The grey dot is a no-relatedness control. Coefficient of relatedness should be 0.5 for a parent-child pair. ibs0: the number of sites at which the 2 samples shared no alleles (should approach 0 for parent-child pairs). ibs2: the number of sites in which the child vs parent samples where both hom-ref, both het, or both hom-alt. Shared_hets: the number of sites at which both child and parent samples were hets. (**b**) Ancestry check using Peddy (proband and parents are purple dots). (**c**) Phylogenetic conservation of TLR7 variants. (**d**) Integrative Genomics Viewer (IGV) image of the Y264H *TLR7 de novo* variant. (**e**) TLR7 structure 6IF5. Regions in red were restrained through all simulations with a harmonic restraint of force constant 5 kcal/mol/Å2, and correspond to residue numbers: 27-96, 116-179, 193-256, 281-297, 304-321, 327-346, 361-376, 385-403, 412-427, 434-460, 476-499, 510-523, 534-548, 561-572, 591-602, 616-625, 646-656, 671-681 and 699-835. (**f**) Guanosine and R848 illustrated with binding geometries from crystal structures 5GMF and 5GMH14. L1-L3 indicate ligand atoms used for Boresch restraints, which were restrained relative to the three depicted protein alpha carbons of residues F408, G379 and F325 (not to scale). Distances and angles in gold, and dihedrals in pink show the values for the 6DoF Boresch restraints. Additional geometric relationships between the restrained atoms, as measured from the starting structure, are shown in grey smaller print. Boresch dihedral restraints are relative to the two atoms connecting either side of the location of print. White hydrogen spheres and red oxygen spheres show the atoms used in the calculation for determining the number of waters within 3.5 Å of the tail region that each ligand interacted with.

We undertook whole-genome sequencing of a Spanish girl who was diagnosed with SLE at the age of 7 (Supplementary Table 1). She first presented with refractory autoimmune thrombocytopenia and had elevated anti-nuclear antibodies (ANAs) and hypocomplementaemia. She went on to develop inflammatory arthralgias, constitutional symptoms, intermittent episodes of hemichorea, and had mild mitral insufficiency and renal involvement after admission with a hypertensive crisis. Bioinformatics analysis revealed a de novo, *TLR7* p.Tyr264His (Y264H) missense variant that was predicted to be damaging by SIFT and CADD (Fig. 1a–c (family A) and Supplementary Table 2). This variant was not present in the databases of normal human genome variation (gnomAD, ExAC, dbSNP). Examination of the BAM files together with paternity analysis confirmed that the mutation occurred de novo (Extended Data Fig. 1a, b, d). The mutated tyrosine residue lies in the eighth leucine-rich repeat of TLR7^25^, within the endosomal part of the receptor (Fig. 1b) and is highly conserved across species, including zebrafish (Fig. 1d). Additional analyses for rare variants in 22 genes that can cause human SLE when mutated (Supplementary Table 3) revealed a heterozygous variant in *RNASEH2B*, p.Ala177Thr, which, when homozygous, causes SLE^26^.

Whole-exome sequencing (WES) analysis of additional patients with SLE identified two other variants in *TLR7* (B.I.2 F507L and C.I.1 R28G; Fig. 1a–d, Extended Data Fig. 1c, Supplementary Tables 1, 2). Notably, in family B the mother had SLE from her mid-twenties and the daughter was diagnosed with neuromyelitis optica in the presence of ANAs and antibodies to aquaporin-4  (AQP4-Ab, also known as NMO-IgG) in the serum and cerebrospinal fluid. Both carried the *TLR7*^*F507L*^ variant, which was also highly conserved (Fig. 1d). No additional rare variants in the 22 SLE-causing genes were identified in these families (Supplementary Table 3).

## *TLR7*^*Y264H*^ increases guanosine sensing

To test whether pathogenic variants enhance TLR7 signalling and downstream NF-κB activation, we transfected RAW264.7 cells with plasmids encoding the different TLR7 mutants. Compared with wild-type cells, overexpression of *TLR7*^*Y264H*^ and *TLR7*^*F507L*^ constructs showed enhanced NF-κB activation for the mutant variants after 2′,3′-cGMP stimulation, whereas overexpression of *TLR7*^*R28G*^ showed enhanced NF-κB activation after stimulation with guanosine and single-stranded RNA (ssRNA) (Fig. 1e, f). Using a published TLR7 structure, we mapped the mutated Tyr264 residue to TLR7 ligand-binding site 1, where R837/R848 and guanosine or its analogues (such as 2′,3′-cGMP) bind^10^. The Tyr264 side-chain OH has been shown to form a hydrogen bond with 2′,3′-cGMP^10^. To further understand whether the Y264H mutation could enhance TLR7 sensing or activation, we used the method of thermodynamic integration within molecular dynamics simulations^27–29^ to determine the relative binding affinity of the ligands to dimeric TLR7 (Extended Data Fig. 1e, f). Guanosine, but not R848, appeared to have increased affinity to itsbinding site with both singly (H264) and doubly protonated (H^+^264) protonated variants (Fig. 1g). The site of the mutation, Tyr264, was close (albeit not bound) to the guanosine, which formed a hydrogen bond to Thr586, limiting the access of water to this end of the ligand (Fig. 1h). By contrast, the mutants H264 and H^+^264, appeared to allow solvent to access this region, helping to stabilize a cluster of water molecules that provide a favourable environment for the polar ribose ring of guanosine (Fig. 1i). In these simulations, Y264H^+^ also displayed stronger attractive electrostatic interactions with guanosine, providing a plausible explanation for the much larger predicted affinity to this variant. The electrostatic attraction of H^+^264 is predicted to be even greater for the natural ligand 2′,3′-cGMP that carries a negative charge.

## *TLR7*^*Y264H*^ causes autoimmunity in mice

**Extended Data Fig. 2:**
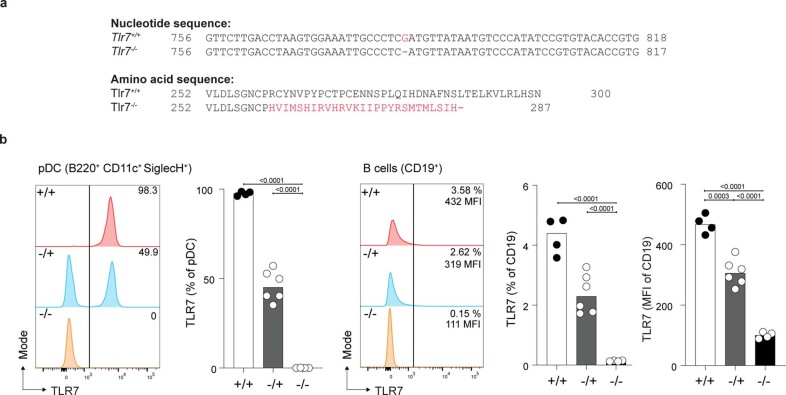
*Tlr7*^−/−^ spleen cells lack TLR7 expression. (**a**) Tlr7 nucleotide and amino acid sequence in mice carrying a CRISPR/Cas9-generated deletion (*Tlr7*^−/−^) and WT littermate (*Tlr7*^+/+^). (**b**) Flow cytometric histograms of intracellular TLR7 expression on cells from 6-month-old mice of the indicated genotypes: plasmacytoid dendritic cells (pDC, CD19^−^ CD11c^+^ SiglecH^+^ BST2^+^) and CD19^+^ B cells. Bars represent medians and each dot a single mouse. These results are representative of one experiment. One-way ANOVA with Tukey (**b**); Exact *p* values are shown.

**Extended Data Fig. 3:**
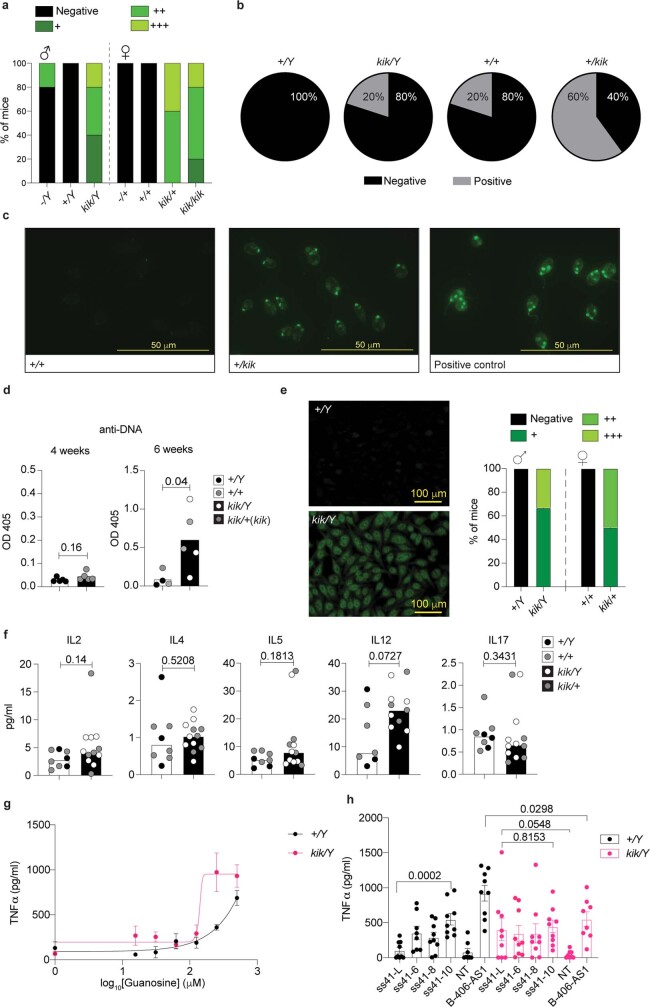
Autoantibodies to ssDNA but not dsDNA are a feature of *kika* mice and are detectable by 6 wks of age. (**a**) Quantification of ANAs in 12 wk-old kika mice by Hep-2 immunofluorescence (IF). (**b**) Proportion of 6 month-old WT and *kika* mice positive for dsDNA according to *Crithidia luciliae* IF, and representative images (**c**) for each genotype. (**d**) Autoantibodies to DNA in serum from 4 wk-old (n = 10) and 6 wk-old (n = 9) wt or *kika* mice. (**e**) HEp-2 IF showing pattern and quantification of ANAs in 6 wk-old *kika* mice. (**f**) Mesoscale measurement of cytokines in serum from wt or *kika* mice (n = 20). (**g**) Dose-dependent response of TLR7 to guanosine (data averaged from two mice in biological triplicate). (**h**) Responsiveness of TLR7 to ssRNAs lacking uridine (ss41-L), with 6 to 10 uridines; or 9 uridines B-406-AS1 (data represent mean ± s.e.m. averaged from three mice in biological triplicates). Unpaired *t*-test (**d**, **h**); Mann-Whitney test (**f**). Exact *p* values are shown.

Our modelling suggested *TLR7*^*Y264H*^ would increase affinity to endogenous ligands. To investigate whether *TLR7*^*Y264H*^ could cause SLE, we introduced the orthologous allele into C57BL/6 mice using CRISPR–Cas9 editing. WES analysis confirmed that *Tlr7*^*Y264H*^ was the only relevant CRISPR-induced coding variant segregating with the phenotype. The resulting strain was named kika and *Tlr7*^*Y264H*^ is hereafter named the *kik* allele. A CRISPR-generated line lacking TLR7 protein owing to a 1-bp deletion was included as a control (Extended Data Fig. 2a, b). Male or female mice (aged 12 weeks) carrying one or two *kik* alleles displayed splenomegaly with increased cellularity (Fig. 1j), decreased survival (Fig. 1k) and ANAs with nuclear, cytoplasmic, cell-cycle-dependent and Golgi staining (Fig. 1l, Extended Data Fig. 3a). These were detected from 6 weeks of age in female kika mice (Extended Data Fig. 3d, e). Male *Tlr7*^*kik/Y*^ and female *Tlr7*^*kik/+*^ kika mice also developed antibodies to the TLR7 ligands ssRNA and Smith protein (Sm) and ribonucleoprotein (RNP), an RNA-containing nuclear self antigen^7^ (Fig. 1m), and 10–20% had weak double-stranded DNA reactivity (Extended Data Fig. 3b, c). These results suggest *TLR7*^*Y264H*^ is an X chromosome-linked dominant GOF allele.

## H264 increases the response to guanosine

To validate the predicted increased affinity of H264 for guanosine, we tested the response of *Tlr7*^*kik/Y*^ kika bone-marrow-derived macrophages (BMDMs) to increasing doses of guanosine and R848. Although no difference was observed with R848 (Fig. 1n and data not shown), we found an increased responsiveness to guanosine from kika BMDMs compared with wild-type BMDMs (Fig. 1o and Extended Data Fig. 3g). Both the first (guanosine-binding) and second (uridine-binding) sites of TLR7 are necessary for ssRNA-induced TLR7 signalling^8,10,12^. We tested the responsiveness of wild-type ands mutant BMDMs to ssRNAs lacking uridine (ss41-L) or containing 6 to 10 uridines. Interestingly, whereas ssRNA sensing by wild-type BMDMs correlated with uridine content, ssRNA sensing by kika BMDMs was independent of uridine content, and ssRNA lacking uridine induced TLR7 activity in kika cells (Extended Data Fig. 3h). These results collectively indicate that the kika mutation selectively increases sensing of guanosine to the first site independently of activity at the uridine-selective second site, therefore raising the sensitivity to otherwise non-TLR7-stimulating ssRNAs.

## Tissue damage caused by *TLR7*^*Y264H*^

**Fig. 2:**
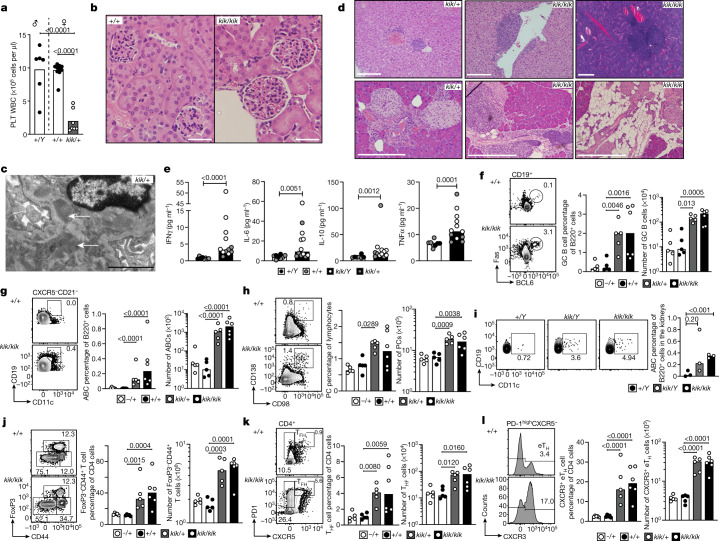
Kika mice develop autoimmune symptoms and end organ damage. **a**, The platelet count in female *Tlr7*^*kik/+*^ mice (aged 18 weeks). **b**, **c**, Haematoxylin and eosin (H&E) staining (**b**) and electron microscopy analysis (**c**) of kidneys from kika and control mice (aged 6 months). The white arrows show immunoglobulin deposits. Scale bars, 50 µm (**b**) and 2 µm (**c**). **d**, H&E staining of the liver, pancreas and salivary gland from kika mice (aged 12–21 weeks). Scale bars, 100 µm (bottom left) and 200 µm (other images). **e**, Mesoscale measurement of cytokines in serum from wild-type (*n* = 8) or kika (*n* = 12) mice. **f**–**l**, Flow cytometry plots and quantification of splenic cells from kika mice (aged 12 weeks): GC B cells (CD19^+^CD95^+^BCL6^+^) (**f**); ABCs (B220^+^CD21^−^CXCR5^−^CD19^high^CD11c^+^) (**g**); plasma cells (PCs; CD138^+^CD98^+^) (**h**); ABCs in the kidneys from kika mice (**i**); CD4 effector T cells (CD4^+^FOXP3^−^CD44^+^) (**j**); T_FH_ cells (CXCR5^+^PD1^high^) (**k**); and extrafollicular helper T cells (eT_H_; CD4^+^CXCR5^−^PD1^+^CXCR3^+^) (**l**). The bars represent the median values, and each dot represents a single mouse. These results are representative of *n* = 4 (**f**–**h**, **j–l**), *n* = 3 (**b**, **d**), or *n* = 2 (**a**, **i**) experiments. Experiments in **c** were performed once with 5 mice (WT *n* = 2, kika *n* = 3) and experiments in **e** were performed once with serum from 20 mice. Statistical analysis was performed using one-way ANOVA with Tukey multiple-comparison test (**a**, **f**–**h**, **j**–**l**); unpaired *t*-tests (**i**); and Mann–Whitney *U*-tests (**e**). The exact *P* values are shown.

**Extended Data Fig. 4:**
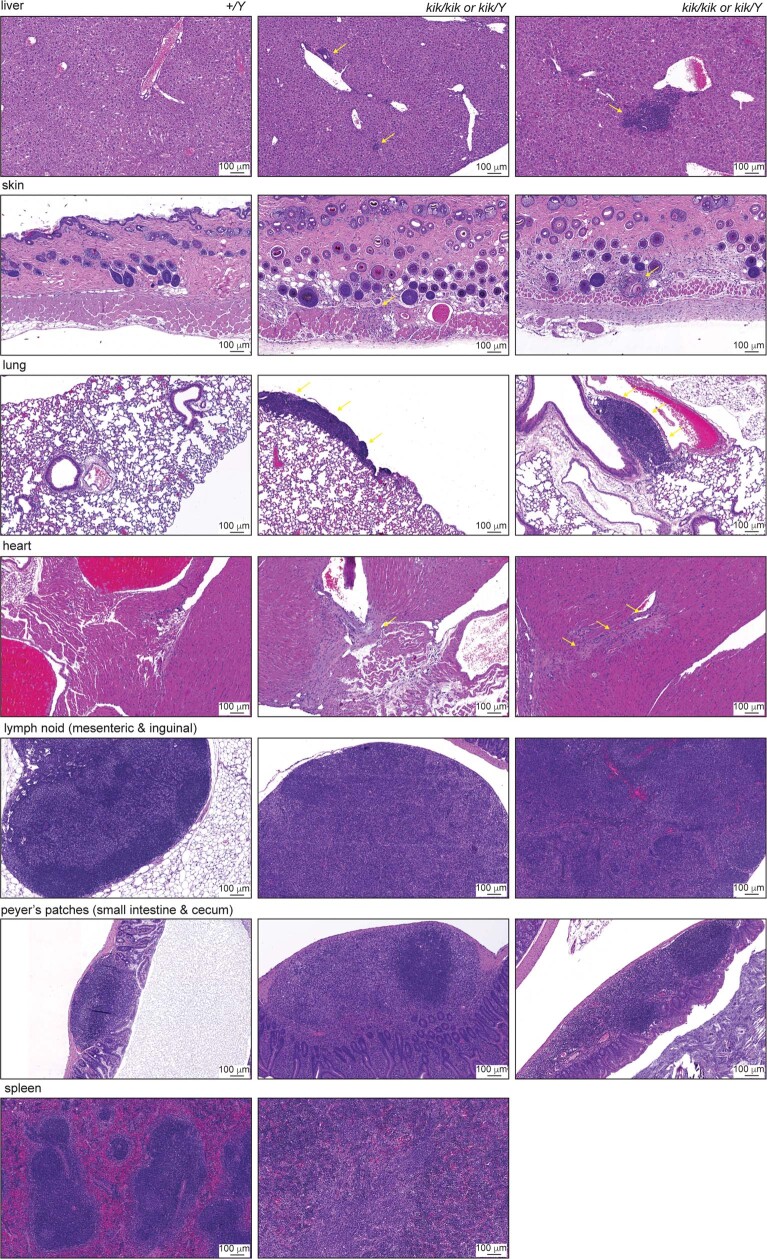
Organ Pathology of *kika* mice. Observational report is summarized in supplementary table 4. Organs were collected from 3 mice per genotype. Histopathology and organ pathology was performed by the Australian Phenomics Network (APN).

**Extended Data Fig. 5:**
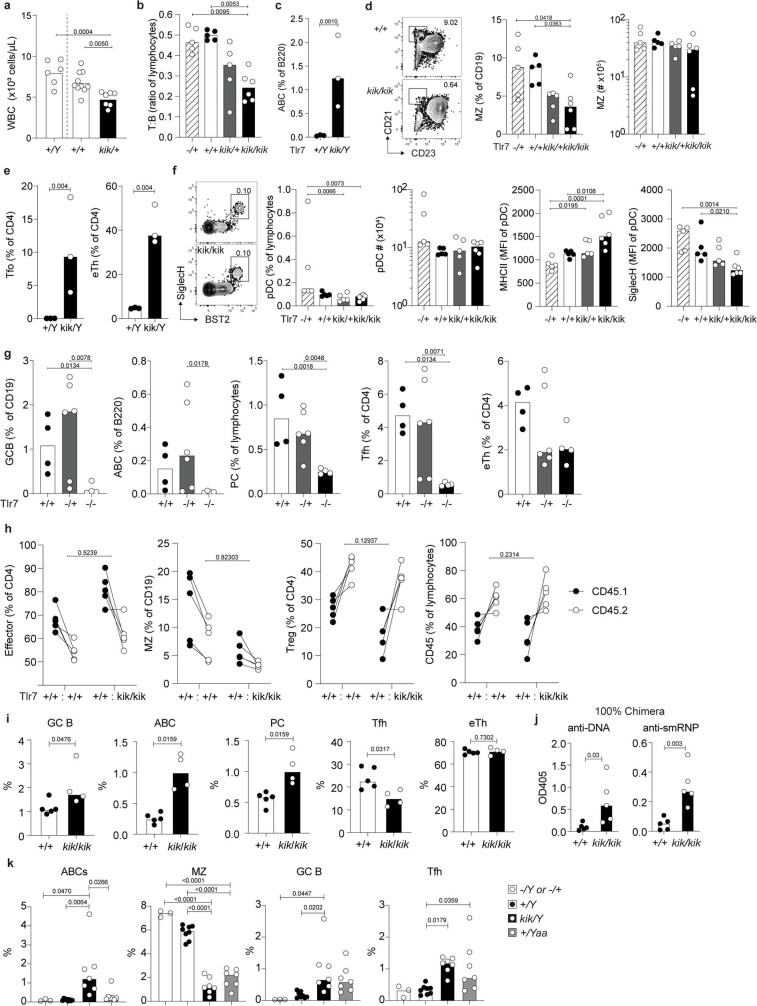
Cellular phenotypes in blood and spleen from *kika* mice, TLR7-deficient mice, mixed chimeras and *Yaa* mice. (**a**) White blood cell (WBC) count in 18-wk old mice. (**b**, **c**) Flow cytometric plots and quantification. (b) Spleen T (CD3^+^):B (B220^+^) cell ratio from 12-wk old *kika* mice. (**c**) Age-associated B cells (ABC, B220^+^ CD21^−^ CD23^−^ CD19^hi^ CD11c^+^) in blood from 18-wk-old *kika* mice. (**d**) Splenic marginal zone (MZ) B cells (CD19^+^ CD23^−^ CD21^+^) in 12-wk *kika* mice. (**e**) Circulating T follicular cells (Tfo, CD4^+^ CXCR5^+^ PD1^hi^) and extrafollicular helper T cells (eT_H_, CD4+ CXCR5^−^ PD1^+^ CXCR3^+^) in blood from 18-wk-old *kika* mice. (**f**) Plasmacytoid dendritic cellc (pDCs, CD3^−^ CD19^−^ MHCII^+^ CD11c^+^ CD11b^-^ CD8^−^ SiglecH^+^ BST2^+^) and pDC MFI of MHCII and SiglecH from 12-week-old *kika* mice. (**g**) Splenic germinal center B cells (GCB, CD19^+^ CD95^+^ BCL6^+^), ABC (B220^+^ CD21^−^ CXCR5^−^ CD19^hi^ CD11c^+^), T_FH_ (CD4^+^ CXCR5^+^ PD1^hi^), eT_H_ (CD4+ CXCR5^−^ PD1^+^ CXCR3^+^) and plasma cells (PC, CD138^+^ CD98^+^) from 24-wk TLR7-deficient mice. (**h**, **i**) Splenic cell subsets from mixed bone marrow chimeric mice containing a 1:1 ratio of control Tlr7^+/+^ CD45.1/Tlr7^+/+^ CD45.2 or Tlr7^+/+^ CD45.1/Tlr7^kik/kik^ CD45.2 bone marrow (**h**) and 100% mixed bone marrow (**i**) of each genotype. Subsets shown are CD4 effector (CD4^+^ FoxP3^−^ CD44^+^), MZ (CD19^+^ CD23^−^ CD21^+^), Treg (CD4^+^ FoxP3^+^) and CD45.1 to CD45.2 reconstitution ratio, 22-weeks post-reconstitution. (**j**) Autoantibodies to DNA and smRNP in serum from 100% chimeric mice model. (**k**) Splenic cell subsets from *kika* and *Yaa* mice. Bars represent medians and each dot a single mouse. These results are representative of one blood ADVIA analysis, two experiments for blood flow cytometry, four splenic phenotyping for *kika* mice and one for TLR7 deletion mice, and one experiment for chimera analysis. One-way ANOVA with Tukey (**a**–**g**, **k**); Two-way ANOVA (**h**); Mann-Whitney (**i**); Unpaired *t*-test (**j**); Exact *p* values are shown.

In-depth phenotyping of kika mice revealed marked thrombocytopenia as seen in the proband (Fig. 2a) and a slightly lower white blood cell count (Extended Data Fig. 5a). Proliferative glomerulonephritis was evident in kidneys (Fig. 2b), as well as expanded mesangial matrix with electron-dense deposits and increased mesangial cellularity (Fig. 2c). Lymphoid infiltrates were seen in the liver, salivary glands and pancreas (Fig. 2d) where they occasionally formed peri-islet follicular structures. Exocrine pancreatic tissue was often replaced by fat, particularly in *Tlr7*^*kik/kik*^ mice (Fig. 2d). Other findings included subpleural, perivascular and interstitial infiltrates in the lungs; myocyte degeneration and necrosis in skin panniculus muscle; focal myocardial fibrosis, splenic lymphomas (4 out of 6 mice); chronic lymphadenitis in the lymph nodes and gut; and hyperplasia of Peyer’s patches (Supplementary Table 4, Extended Data Fig. 4). Serum levels of IFNγ, IL-6, IL-10 and TNF were increased (Fig. 2e, Extended Data Fig. 3f).

Flow cytometry analysis of kika spleens revealed a reduced T:B cell ratio with an increase in total B cells, spontaneous GCs, and increased plasma cells and ABCs; ABCs were also expanded in the blood and kidneys (Fig. 2f–i, Extended Data Fig. 5b, c). The percentages of splenic marginal zone B cells were decreased, but the total numbers were not decreased (Extended Data Fig. 5d). Effector or memory CD4^+^CD44^high^ cells, including T_FH_ cells and CXCR3^+^ extrafollicular helper CD4^+^ T cells, were increased in kika mice (Fig. 2j–l) as well as circulating CXCR5^high^PD-1^high^ GC T_FH_ cells that are usually only seen in secondary lymphoid tissues (Extended Data Fig. 5e). Plasmacytoid dendritic cells (pDCs) appeared to be activated with increased MHC-II and reduced siglec-H expression^30^ (Extended Data Fig. 5f). TLR7-deficient mice lacked spontaneous GC B cells, T_FH_ cells and ABCs, and had reduced plasma cells (Extended Data Fig. 5g), supporting that the expansion of these subsets in kika mice is driven by the TLR7 GOF.

## B-cell-intrinsic effects of *TLR7*^*Y264H*^

**Fig. 3:**
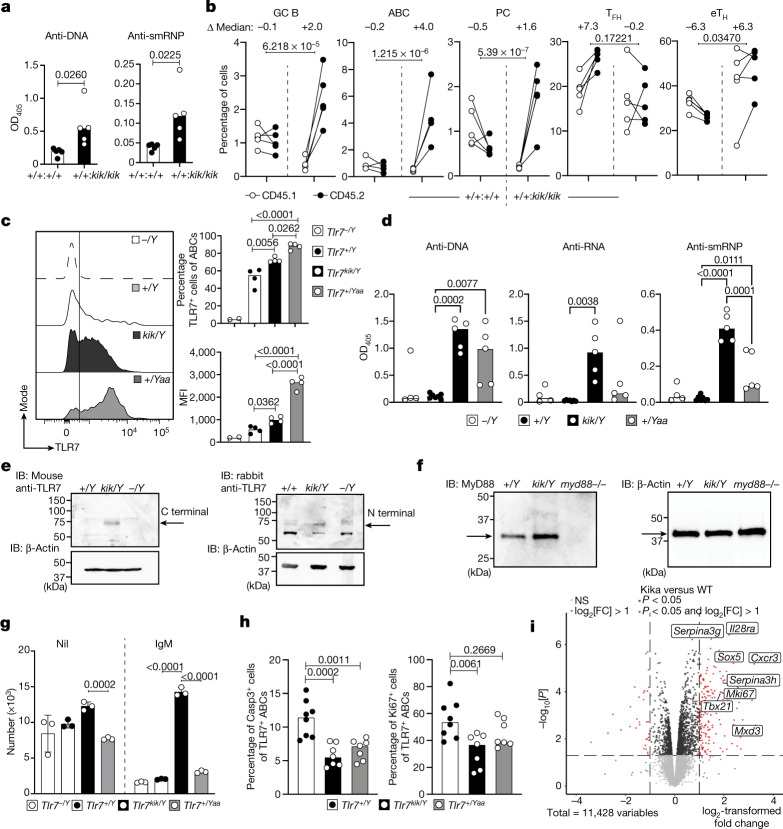
Cell-intrinsic expansion of ABCs and GC B cells in kika mice, aberrant B cell survival and extrafollicular autoimmunity induced by the Y264H variant. **a**, **b**, Autoantibodies to DNA and smRNP in the serum (**a**) and cellular splenic phenotypes (**b**) from mixed bone marrow chimeric mice containing a 1:1 ratio of control *Tlr7*^+/+^CD45.1/*Tlr7*^+/+^ CD45.2 or *Tlr7*^+/+^CD45.1/*Tlr7*^*kik/kik*^CD45.2 bone marrow. **c**, Histogram plot and quantification of TLR7 expression in ABCs from *Tlr7*^*−/Y*^ (*n* = 2),* Tlr7*^*+/Y *^(*n* = 4),* Tlr7*^*kik/Y*^ (*n* = 4) and *Tlr7*^*+/Yaa*^ (*n* = 4) mice. **d**, Autoantibodies to DNA, RNA and smRNP in the serum from *Tlr7*^*−/Y*^ (*n* = 4), *Tlr7*^*+/Y*^(*n* = 7), *Tlr7*^*kik/Y*^(*n* = 5) and *Tlr7*^*+/Yaa*^ (*n* = 5) mice. **e**, **f**, Western blot analysis showing splenocyte expression of TLR7 (**e**) and MyD88 (**f**) from mice of the indicated genotypes. IB, immunoblot. **g**, Survival of magnetic-activated cell sorting (MACS)-purified splenic B cells cultured with or without anti-IgM for 72 h from male mice of the indicated genotypes. Data are mean ± s.d. **h**, Quantification of apoptosis (caspase-3) and proliferation (Ki67) in ABCs from male mice (*Tlr7*^*+/Y*^ *n *= 8,  *Tlr7*^*kik/Y*^ *n* = 7, *Tlr7*^*+/Yaa*^ *n* = 7). **i**, Differentially expressed genes in MACS-purified splenic B cells from wild-type (*n* = 3) or kika (*n* = 3) mice cultured with anti-IgM (10 µg ml^−1^) for 20 h. The bars represent the median values and each dot represents a single mouse. These results are representative of *n* = 2 (**c**, **e**, **f**, **h**)  or *n* = 3 (**d**, **g**) independent experiments. Experiments in **a**–**e** were performed once with *n* > 25 mice, and experiments in **j**–**n** were performed once with *n* = 4 HC-teens, *n* = 3 HC-adults and *n* = 1 patient. Statistical analysis was performed using unpaired *t*-tests (**a**); one-way ANOVA with Tukey multiple-comparison test (**c**, **d**, **h**); and two-way ANOVA with Tukey multiple-comparison test (**b**, **g**). Exact *P* values are shown. NS, not significant.

To establish which phenotypes were cell autonomous, mixed bone marrow chimeras were generated by adoptively transferring 100% wild-type CD45.2 or kika CD45.2 bone marrow, or 50:50 mixes of either wild-type CD45.1:kika CD45.2 or wild-type CD45.1:wild-type CD45.2 bone marrow into sublethally irradiated *Rag1*^*−/−*^ mice (Fig. 3a, b, Extended Data Fig. 5h). Autoantibodies were present in chimeric mice receiving either 100% or 50% kika bone marrow cells (Fig. 3a, Extended Data Fig. 5j). Expansion of GCs, ABCs and plasma cells (Fig. 3b) was cell-intrinsic whereas all T cell phenotypes and reduction in marginal zone B cells were largely cell-extrinsic (Fig. 3b, Extended Data Fig. 5h, i). The ABCs of kika mice expressed more TLR7 although not to theextent seen in Yaa mice that express two copies of *Tlr7* (Fig. 3c). By contrast, the functional consequences of the Y264H mutation were more severe than the *Yaa* allele, as seen by higher levels of anti-RNA and smRNP autoantibodies and ABCs in kika mice (Fig. 3d, Extended Data Fig. 5k).

We investigated whether the Y264H variant leads to spontaneous TLR7 cleavage and activation in the absence of stimulation^12^. Western blot analysis of splenocyte lysates from kika and wild-type littermates using two different antibodies against both the C and N termini revealed the presence of the approximately 75-kDa C-terminal-cleaved and 65-kDa N-terminal cleaved TLR7 product in splenocytes from unimmunized kika mice (Fig. 3e). Such cleaved fragments have been reported to be indicative of the active form of TLR7^31^. MyD88 was also increased in kika splenocytes (Fig. 3f), consistent with enhanced TLR signalling^32^.

**Extended Data Fig. 6:**
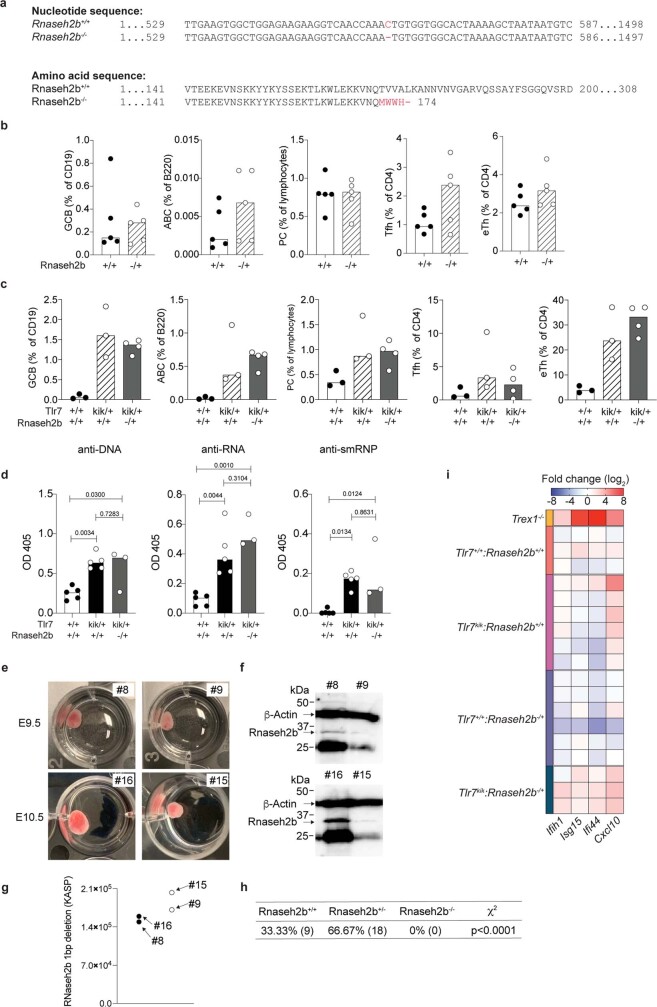
*Rnaseh2b* hemizygosity does not cause a cellular phenotype. (**a**) *Rnaseh2b* cDNA sequence from *Rnaseh2b*-deletion mice, highlighting the single nucleotide CRISPR/Cas9-generated deletion leading to a frame-shift after amino acid residue Q170 and stop codon 4 amino acids downstream. (**b**, **c**) Flow cytometric quantification of splenic germinal center B cells (GCB, CD19^+^ CD95^+^ BCL6^+^), age-associated B cells (ABC, B220^+^ CD21^−^ CXCR5^−^ CD19^hi^ CD11c^+^), T follicular helper cells (T_FH_, CD4^+^ CXCR5^+^ PD1^hi^), extrafollicular helper T cells (eT_H_, CD4^+^ CXCR5^−^ PD1^+^ CXCR3^+^) and plasma cells (PC, CD138^+^ CD98^+^) (**b**) in 12 week-old mice carrying a heterozygous deletion in *Rnaseh2b* and (**c**) in 12-wk-old mice with heterozygous deletion in *Rnaseh2b* crossed to the *kika* mice. (**d**) Autoantibodies to DNA, RNA and smRNP in serum from mice of indicated genotypes. (**e**) Representative photo of time-mated embryos from *Rnaseh2b*^−/+^ breeders. (**f**) Western blot of RNASEH2B in time-mated embryo lysates. (**g**) KASP genotyping results of representative time-mated embryos. (**h**) Breeding record of *Rnaseh2b*^−/+^ crossed to *Rnaseh2b*^−/+^ mice. (**i**) Type 1 IFN signature of *Rnaseh2b*^*+/−*^ *Tlr7*^*kik/+*^ double heterozygous female mice compared to *Tlr7*^*kik/+*^ alone, *Rnaseh2b*^*+/−*^ alone or *Trex1*^*−/−*^ mice as positive controls. Bars represent medians and each dot a single mouse. Data is representative of two experiments. Embryos were collected from two time-mating breeding set ups (n = 18 embryo collected from two pair breeders). One-way ANOVA with Tukey (**b**–**d**); *Chi*-square test (**h**); Exact *p* values are shown.

Proband A.II.1 was also heterozygous for *RNASEH2B* p.Ala177Thr, which, when homozygous, causes SLE and Aicardi–Goutières syndrome^26^. RNASHE2B activates cGAS–STING, which increases type 1 IFN production and TLR7 signalling^33^. To test whether this allele might act in functional epistasis with *TLR7*^*Y264H*^, we generated mice carrying an *Rnaseh2b* 1 bp deletion, leading to a premature stop codon at amino acid 175 (Extended Data Fig. 6a). Heterozygous mice were viable and had no immunological phenotypes (Extended Data Fig. 6b), whereas the mutation was embryonically lethal in homozygous mice, as previously reported for *Rnaseh2b-*knockout mice^34^ (Extended Data Fig. 6e–h). Besides a mild enhancement in the expression of type I IFN signature genes (Extended Data Fig. 6i), *Rnaseh2b* hemizygosity did not exacerbate the cellular or serological phenotypes of *Tlr7*^*kik/+*^ female mice (Extended Data Fig. 6c, d). However, it is possible that there may be functional epistatic effects of these two RNA-interacting proteins in the presence of environmental triggers.

## Enhanced survival of BCR-activated cells

**Extended Data Fig. 7:**
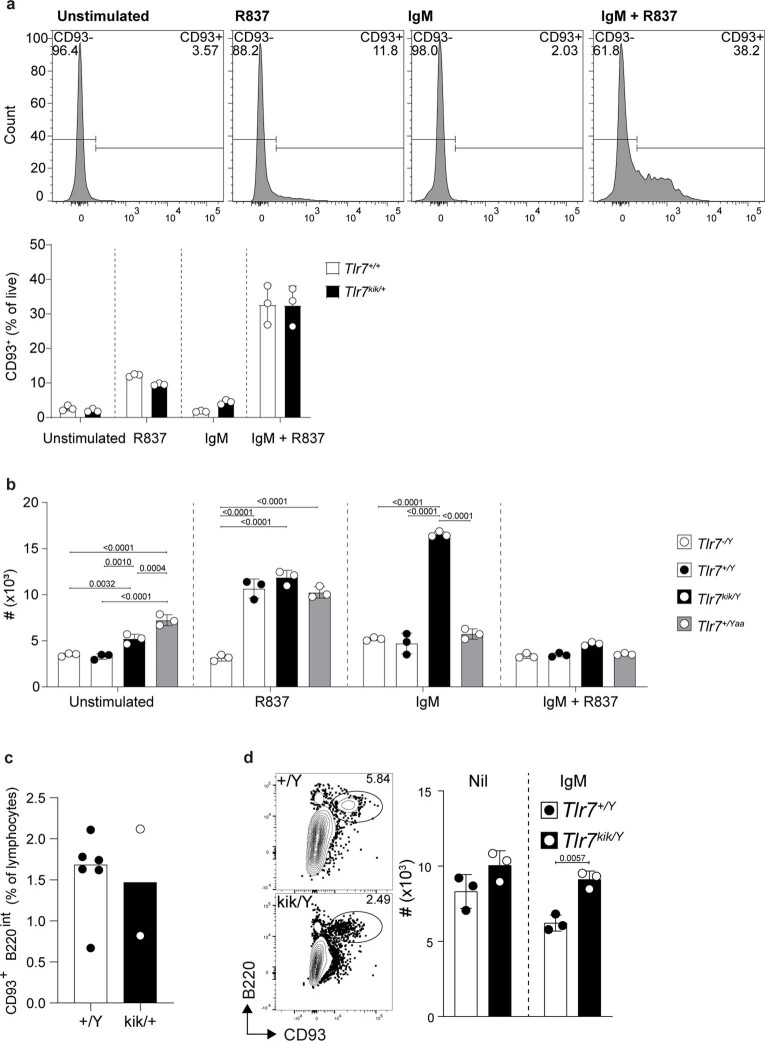
*Kika* mice have normal responses to TLR7 signalling and CD93 is expressed on TLR7 stimulated B cells. (**a**–**c**) Flow cytometric analysis of (**a**) Mean CD93^+^ cells derived from CD93^−^ sorted splenic B cells stimulated with R837, a-IgM or R837 + a-IgM for 72-hours from *kika* and control mice. (**b**) FACS sorted bone marrow immature B cells (B220^int^ CD93^+^) cultured with or without α-IgM for 72 h, from male mice of the indicated genotypes. (**c**) Mean survival count of MACS purified splenic B cells stimulated with R837, a-IgM or R837 + a-IgM for 72-hours from *kika* mice and control mice. (**c**) Percentage of bone marrow (BM) immature B cells (CD93^+^ B220^int^) in 12-35-wk *kika* and control mice. (**a**, **b**, **d**) Bars represent means ± s.d. and (**d**) each dot a single mouse. These results are representative of one experiment for CD93 upregulation and BM immature flow cytometry, four splenic B cell cultures purified using MACS bead selection and one BM analysis. Two-way ANOVA with Tukey (**a**, **b**) and Sidak (**d**); Exact *p* values are shown.

We next examined the stage at which *TLR7*^*Y264H*^ breaks B cell tolerance. We hypothesized that constitutive TLR7 signalling may provide an aberrant signal 2 to self-reactive B cells that have bound to self-antigen through their BCR (signal 1) and would otherwise die within 72 h, as occurs in anergic B cells^35^ and in immature CD93^+^ B cells stimulated with anti-IgM^36^. We could not use CD93 to purify immature splenic B cells because we found that agonistic TLR7 treatment of mature B cells upregulated CD93 (Extended Data Fig. 7a). We therefore activated either total splenic B cells or CD93^+^B220^low^ immature bone marrow cells from kika, wild-type and Yaa mice carrying a *Tlr7* duplication^6,7^, stimulated them with R837 or anti-IgM and performed live cell counts 72 h later. We observed that anti-IgM, but not R837, enhanced the survival of total, mature and immature kika B cells compared with control cells (Fig. 3g, Extended Data Fig. 7b–d). This differs from reports using TLR7 transgenic B cells, which displayed increased survival only when activated with a TLR ligand and not with anti-IgM^37^, again suggesting sustained activation of TLR7(Y264H) by endogenous ligands. RNA-sequencing (RNA-seq) analysis of kika and control splenocytes cultured for 20 h with anti-IgM revealed that 203 and 34 transcripts were upregulated or downregulated, respectively, by more than twofold (*P* < 0.05) in kika cells (Fig. 3i). Upregulated transcripts included the anti-apoptotic genes *Mxd3*^38^ and *Serpina3g*^39^, as well as *IL28ra* (also known as *Ifnlr1*), which encodes the common IFNλ-1/2/3 receptor^40^. Levels of the transcription factor SOX5, which decreases B cell proliferative capacity while allowing plasmablast differentiation, were also^41^. Other upregulated transcripts included *Cxcr3*, which promotes lupus nephritis^42^. We confirmed a decreased tendency for apoptosis in kika ABCs with decreased expression of active caspase-3 and also a small decrease in proliferation (Fig. 3h). Overall, these results suggest that hypersensitive TLR7 signalling enables the survival of B cells that bind to self-antigen through their surface BCR.

## MyD88 dependence and GC independence

**Fig. 4:**
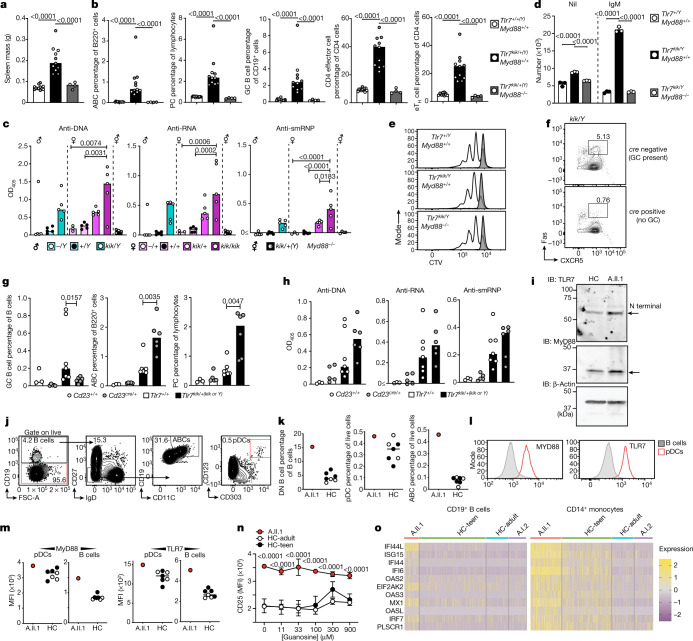
TLR7-mediated autoimmunity is MyD88 dependent. **a**, **b**, Spleen mass (**a**) and flow cytometry quantification (**b**) of ABCs (B220^+^CD21^−^CXCR5^−^CD19^high^CD11c^+^), plasma cells (CD138^+^CD98^+^), GC B cells (CD19^+^CD95^+^BCL6^+^), CD4 effector/memory T cells (CD4^+^FOXP3^−^CD44^+^) and extrafollicular helper cells (CD4^+^CXCR5^−^PD1^+^CXCR3^+^) from splenocytes of male (grey) and female (white) kika mice (aged 12 weeks) either sufficient or deficient in MyD88. **c**, Auto-antibodies to ssDNA (ANAs), ssRNA and smRNP from kika mice (aged 12 weeks) either deficient or sufficient in MyD88. **d**, **e**, Survival (**d**) and proliferation (**e**) of CTV-labelled splenic B cells cultured for 72 h with anti-IgM (white) or unstimulated (grey). **f**, **g**, Flow cytometry plots (**f**) and quantification (**g**) of splenic GC B cells (CD19^+^CD95^+^CXCR5^+^), ABCs and plasma cells from kika or control littermates (aged 10 weeks) either deficient (*Bcl6*^*flox/flox*^;*Cd23*^*cre*^) or sufficient (*Bcl6*^*flox/flox*^) in GCs (*Cd23*^*+/+*^ *n* = 3, *Cd23*^*cre/+*^ *n* = 5, *Tlr7*^*+/+*^ *n* = 7,* Tlr7*^*kik/+*^^*(kik or Y)*^*n *= 6). **h**, Serum autoantibodies to ssDNA, ssRNA and smRNP in the same mice as in **g**. **i**, Western blots showing TLR7 and MyD88 expression in PBMCs from A.II.1 and an age- and gender-matched healthy control individual (HC). **j**, **k**, Flow cytometry plots (**j**) and quantification (**k**) of the DN B cell, pDC and ABC phenotype in PBMCs from healthy control individuals (*n* = 7) and A.II.1. **l**, Quantification histograms of TLR7 and MyD88 protein in healthy control individuals and A.II.1. **m**, Mean fluorescence intensity (MFI) of MyD88 and TLR7 protein expression in plasmacytoid dendritic cells (pDC) and B cells in healthy control individuals and A.II.1. **n**, Quantification of CD25 expression in CD14^+^ monocytes after dose stimulations with guanosine. Data are mean ± s.d. **o**, Type I IFN signature of healthy control individuals (HC-teen and HC-adult), A.I.2 and A.II.1. The bars represent the median values and each dot represents a single mouse. The results represent *n* = 2 (**f**–**i**), or *n* = 1 (**a**–**e**, **k**–**o**), independent experiments. Experiments in **a**–**e** were performed once with *n* > 25 mice, and experiments in **j**–**n** were performed once with *n* = 4 HC-teens, *n* = 3 HC-adults, *n* = 1 patient. Statistical analysis was performed using one-way ANOVA with Tukey multiple-comparison test (**a**–**c**); Mann–Whitney *U*-tests (**g**); and two-way ANOVA with Tukey multiple-comparison test (**d**, **n**). Exact *P* values are shown.

**Extended Data Fig. 8:**
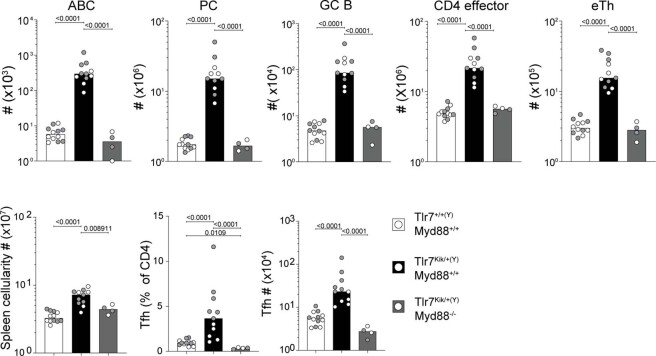
*Myd88* deficiency rescues *kika’s* immune cell phenotype and splenic cellularity. (**a**) Flow cytometric quantification of splenic cell subsets (percentage and total number) and total cellularity in 12 wk-old mice of the indicated genotypes (*Tlr7*^+/+(Y)^*Myd88*^+/+^ n = 12, *Tlr7*^kik/+(Y)^*Myd88*^+/+^ n = 11, *Tlr7*^kik/+(Y)^*Myd88*^−/−^ n = 4). Subsets include: germinal center B cells (GCB, CD19^+^ CD95^+^ BCL6^+^), T follicular helper cells (T_FH_, CD4^+^ CXCR5^+^ PD1^hi^), percentage of ABC (B220^+^, CD21^−^, CXCR5^−^ CD19^hi^ CD11c^+^), extrafollicular helper cells (eT_H_, CD4^+^ CXCR5^−^ PD1^+^ CXCR3^+^), plasma cells (PC, CD138^+^ CD98^+^), CD4 effector/memory T cells (CD4^+^ FoxP3^−^ CD44^+^) Male mice = grey and female mice = white. Bars represent medians and each dot a single mouse. One-way ANOVA with Tukey; Exact *p* values are shown.

To confirm that the observed aberrant B cell survival after IgM stimulation was due to enhanced TLR7 signalling, we crossed kika mice with *Myd88*-knockout mice. MyD88 deficiency completely rescued kika phenotypes, including splenomegaly (Fig. 4a), accumulation of ABCs, GC B cells, plasma cells, eT_H_ cells (Fig. 4b, Extended Data Fig. 8) and autoantibody formation (Fig. 4c). The aberrant survival of B cells receiving only signal 1 was completely abrogated in anti-IgM-activated kika B cells lacking MyD88 (Fig. 4d) without changes in proliferation (Fig. 4e).

It remains controversial whether the spontaneous GCs of lupus-prone mice contribute to the autoimmune phenotype, with some suggesting that TLR7 promotes the appearance of self-reactive GC B cells that produce autoantibodies^17^ and others proposing that the pathogenic B cells are ABCs of extrafollicular origin^1^. To resolve this question, we crossed kika mice with *Bcl6*^*flox/flox*^;*Cd23*^*cre*^ mice that cannot form GCs. In the F_2_-intercross offspring, we enumerated GC B cells and confirmed that kika *Bcl6*^*flox/flox*^;*Cd23*^*cre*^ mice had a substantial reduction in GC B cells (Fig. 4f). Despite the paucity of GC B cells, kika *Bcl6*^*flox/flox*^;*Cd23*^*cre*^ mice developed autoantibodies and an even more pronounced expansion of ABCs and plasma cells than that observed in their GC-forming kika littermates (Fig. 4g, h). These results support the idea that TLR7-driven autoimmunity is GC independent.

**Extended Data Fig. 9:**
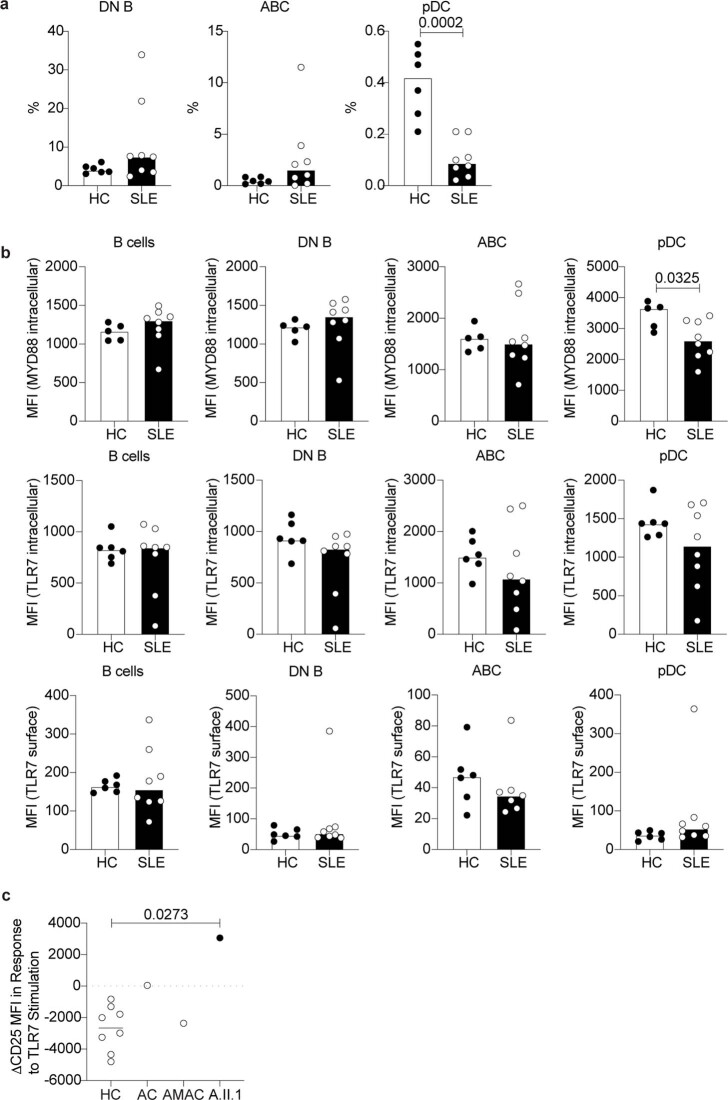
TLR7 and MYD88 expression is indistinguishable between female patients with SLE and healthy controls (HC). (**a**) Flow cytometric analysis and quantification of DN B cells, pDCs and ABCs phenotype in PBMCs from healthy controls (n = 6) and female patients with SLE (n = 8). (**b**) Quantification of TLR7 and MYD88 protein in HC and SLE. (**c**) Quantification of CD25 expression after TLR7 stimulation among HC (n = 8), autoimmune control (AC) (n = 1), age-matched autoimmune control (AMAC) (n = 1) and A.II.1 (n = 1). Unpaired *t*-test (**a**, **b**); One-way ANOVA with Tukey (**c**); Exact *p* values are shown.

We finally analysed the PBMCs from proband A.II.1 carrying the Y264H variant to confirm the phenotypes observed in the kika mice. Flow cytometry analysis revealed that ABCs and the parental IgD^−^CD27^−^ B cells were substantially increased compared with gender- and age-matched controls (Fig. 4j, k). As seen in kika mice, TLR7 and MyD88 protein expression were increased in the pDCs and B cells of the proband (Fig. 4l, m), as was the cleaved TLR7 product (Fig. 4i). Increased levels of ABCs, TLR7 and MyD88 were not seen in the most unrelated patients with SLE (Extended Data Fig. 9a, b). PBMCs in proband A.II.1 also revealed upregulation of the NF-κB activation marker CD25 in unstimulated monocytes (Fig. 4n), increasing its expression after TLR7 stimulation (Extended Data Fig. 9c). The proband with the *TLR7*^*Y264H*^ allele, but not the mother (A.1.2), carrying the *RNASEH2B*^*A177T*^ variant allele (Fig. 4o), had an increased type I IFN signature.

We conclude that TLR7 GOF can cause B-cell-driven autoimmunity including SLE due to increased affinity to guanosine, leading to a lowered threshold for TLR7 activation. Although the human *TLR7*^*Y264H*^ variant is sufficient to induce lupus in mice with no clear additive effects of *Rnaseh2b* hemizygosity apart from increased type I IFN gene transcripts, an exacerbating role of this variant in humans may occur in the presence of environmental stimuli, including ssRNA viruses such as SARS-CoV-2 that are dependent on TLR7 immunity^43^. The TLR7 GOF promotes the survival of BCR-activated immature B cells, which are known to be enriched in self-reactivity^44^. Activation of self-reactive B cells by self-antigen in the presence of a constitutive signal 2 is likely to promote differentiation and autoantibody production of B cells that would otherwise be destined to die in the absence of T cell help.

Notably, despite enhanced TLR7 signalling causing the cell-autonomous accumulation of ABCs and GC B cells, GC B cells are dispensable for the autoimmune phenotype and rather protect against it. Extrafollicular ABCs are therefore the most likely source of pathogenicity. It will be important to determine whether this is true for all cases of SLE, or only for patients in whom excessive TLR7 signalling is the dominant pathogenic pathway. Although highly damaging TLR7 GOF mutations are rare, our data, together with evidence of increased TLR7 signalling in a large fraction of patients with SLE^1^, suggest that TLR7 is a key upstream driver of human SLE. Therapies blocking TLR7 itself or MyD88 may be more effective than therapies blocking GCs in patients with SLE due to increased TLR7 signalling.

## Methods

### Mice

Mice were bred and maintained in specific-pathogen-free conditions at the Australian National University (ANU), Canberra, Australia. Experimentation was performed according to the regulations approved by the local institution ethics committee, including the Australian National University’s Animal and human Experimentation Ethics Committee. Estimations of the expected change between experimental and control groups allowed the use of power analysis to estimate the group size that would enable detection of statistically significant differences. For in vitro experiments, randomization was not required given that there were no relevant covariates. Blinding was used for microscopy: histological analysis, electron microscopy imaging. Mice were used from 6–12 weeks, except for survival curves and tissue assessment (12–26 weeks). Both male and female mice were used and their genders are indicated in most figures (the Y chromosome is indicated in the genotype, that is, male mice).

### Generation of the *Tlr7-* and *Rnaseh2b*-mutant mouse strains

*Tlr7*^*Y264H*^ and deficient mice as well as *Rnaseh2b*-deficient knockout mice were generated in a C57BL/6NCrL background using CRISPR–Cas9-mediated gene editing technology^45^. Genomic sequences were obtained from Ensembl (https://ensembl.org/) and compared to ascertain the conservation of the sequences between mouse and human genes. Single guide RNA (sgRNA) and single-stranded oligonucleotides were purchased from Integrated DNA Technology with the following sequences: *Tlr7*^*Y264H*^ sgRNA, 5′-TATGGGACATTAT*A*ACATCG-3′ with a 5′-AGG-3′ PAM; *Rnaseh2b* 5′-CTTTTAGTG*C*CACCACAGTT-3′ with a 5′-TGG-3′ PAM; *Tlr7*^*Y264H*^ single-stranded oligonucleotide: 5′- GTCAATGAATTGAAAGCATTGTCATGGATCTGTAAGGGGGAATTATTTTCACACGGTGTACACGGATATGGGACATTATGACATCG**AGG**GCAATTTCCACTTAGGTCAAGAACTTGCAACTCATTGAGGTTATTAAAATCATTTTCTTGGATTTTCTTAAT-3′. The italicized nucleotides in the sgRNA sequences indicate the base altered by the respective variant in *Tlr7* or *Rnaseh2b*.

C57BL/6Ncrl female mice (aged 3–4 weeks) were mated with C57BL/6Ncrl males. Pseudopregnant CFW/crl mice were superovulated and mated with stud males. After detection of a vaginal plug, the fertilized zygotes were collected from the oviduct and Cas9 protein (50 ng µl^−1^) was co-injected with a mixture of sgRNA (2.5 ng µl^−1^) and single-stranded oligonucleotides (50 ng µl^−1^) into the pronucleus of the fertilized zygotes. After the micro-injection of the eggs, the zygotes were incubated overnight at 37 °C under 5% CO_2_ and two-cell stage embryos were surgically transferred into the uterine horn of the pseudopregnant CFW/Crl mice. The primers designed to amplify these regions are as follows: Tlr7*-*Y264H-F, 5′-TGAAACACTCTACCTGGGTCA-3′; Tlr7*-*Y264H-R, 5′-GCCTCCTCAATTTCTCTGGC-3′; Rnaseh2b-F, 5′-GCAAGACCATCCCTACTCCA-3′;and Rnaseh2b-R,5′-AACACCTGCCCACATCTGTA-3′.

### Human WES and variant identification

Written informed consent was obtained as part of the Centre for Personalised Immunology Program. The study was approved by and complies with all relevant ethical regulations of the Australian National University and ACT Health Human Ethics Committees, the University Hospitals Institutional Review Board, or by the Renji Hospital Ethics Committee of Shanghai Jiaotong University School of Medicine. For WES analysis, DNA samples were enriched using the Human SureSelect XT2 All Exon V4 Kit and sequenced using the Illumina HiSeq 2000 (Illumina) system. Bioinformatics analysis was performed at JCSMR, ANU as previously described^45^. A search for ‘de novo’, coding, novel or ultrarare (MAF < 0.0005) variants among 100 SLE trios identified a proband with a de novo, novel variant in *TLR7* (Y64H) (family A). A further search for rare variants (MAF < 0.005) in *TLR7* across our three systemic autoimmune cohorts (Australia, Europe and China) that have undergone WES at our Centre for Personalised Immunology (~500 probands) identified 2 additional probands (families B–C). A further proband was identified at Baylor-Hopkins Center for Mendelian Genomics, where the family was recruited as a part of a study investigating monogenic causes of neuroimmune disorders in families with early disease onset (≤10 years). All family members provided written informed consent under Baylor College of Medicine Institutional Review Board (IRB) protocol H-29697. A detailed description of the exome sequencing approach, data processing, filtration and analysis for that particular family can be found in the supplementary information of ref. ^46^. All probands were subsequently analysed for rare variants in 22 genes proven to cause human SLE (Supplementary Table 1).

### Human PBMC preparation

PBMCs were isolated using Ficoll-Paque (GE Healthcare Life Sciences) gradient centrifugation and frozen in fetal bovine serum (FBS, Gibco) with 10% DMSO (Sigma-Aldrich).

### Flow cytometry

Single-cell suspensions were prepared from mouse spleens or thawed PBMCs, and individual subsets were analysed using flow cytometry. The primary antibodies used for mouse tissues included: SiglecH-APC (551, BioLegend), IgD-FITC (405718, BioLegend), IgD-PerCP Cy5.5 (11-26c.2a, BD Pharmingen), CD3-A700 (17A2, BioLegend), CD19-BUV395 (1D3, BD Horizon), CD138-PE (281-2, BD Pharmingen), PD1-BV421 (29F.1A12, BioLegend), CCR7-PerCP Cy5.5 (4B12, BioLegend), CD8-BUV805 (53-6.7, BD Horizon), CD19-BV510 (6D5, BioLegend), CD4-BUV395 (6K1.5, BD Horizon) CD21/35-BV605 (7G6, BD Horizon), CD45.1-BV605 (A20, BioLegend), CD45.1-BV711 (A20, BioLegend), CD45.1-PB (A20, BioLegend), TLR7-PE (A94B10, BD Pharmingen), CD23-BV421 (B3B4, BioLegend), CXCR3-PE (CXCR3-173, BioLegend), CD19-A700 (eBio1D3, Invitrogen), FOXP3-FITC (FJK-16s, Invitrogen (eBioscience), FOXP3-PECy7 (FJK-16s, Invitrogen eBioscience), IgM-FITC (II/41, BD Pharmingen), IgM-PECy7 (II/41, Invitrogen), CD44-FITC (IM7, BD Pharmingen), CD44-PB (IM7, BioLegend), CD95 (FAS)-BV510 (Jo2, BD Horizon), BCL6-A467 (K112-91, BD Pharmingen), CD11b-PerCP Cy5.5 (M1/70, BioLegend), IA/IE-BV421 (M5/114.15.2, BioLegend), CD11c-A647 (N418, BioLegend), CD11c-BV510 (N418, BioLegend), CD11c-FITC (N418, BioLegend), CD25-PE (PC62, BioLegend), B220-A647 (RA3-6B2, BD Pharmingen), B220-BUV395 (RA3-6B2, BD Horizon), B220-BUV737 (RA3-6B2, BD Horizon), CD98-PECy7 (RI.388, BioLegend), CD4-PECy7 (RM4-5, BD Pharmingen), CD25-A647 (PC61, BioLegend), CD4-A647 (RM4-5, BioLegend), CD11c-APC (HL3, BD Pharmingen), CD138-Biotin (281-2, BD Bioscience), CXCR5-Biotin (2G8, BD Bioscience), streptavidin-BUV805 (BD Horizon), streptavidin-BV510 (BioLegend), CD19-BV605 (6D5, BioLegend), B220-PE (RA3-6B2, BioLegend), BST2-PE (927, BioLegend), CD19-PE (6D5, BioLegend), IgD-PE (11-26c.2a, BioLegend), CD11b-PECy7 (M1/70, eBiosciences), streptavidin-PECy (eBiosciences), CD4-PerCPCy5.5 (RM4-5, BioLegend), CD45.2-PerCPCy5.5 (104, BD Bioscience), CD3-Pacific Blue (HIT2, BD Pharmingen). For human PBMCs: CD19-BV650 (HIB19, BioLegend), HLA-DR-BV510 (L243, BioLegend), CD24-BV605 (ML5, BioLegend), CD56-PECy7 (NCAM16.2, BD Pharmingen), CD14-PerCP (MΦP9, BD Pharmingen), IgD-BV510 (IA6-2, BioLegend), CD123-PE (7G3, BD Pharmingen), CD21-APC (B-ly4, BD Pharmingen), CD11c-APC (B-ly6, BD Pharmingen), CD16-APC-H7 (3G8, BD Pharmingen), IgG-PECy7 (G18-145, BD Pharmingen), CD10-PE-CF594 (HI10a, BD Pharmingen), IgA-PE (IS11-8E10, Miltenyi Biotech), CD27-APC-EF-780 (O323, eBiosciences), IgM-EF450 (SA-DA4, eBiosciences), CD38-PerCP-Cy5.5 (A60792, Beckman Coulter), CD93-PECy7 (AA4.1, BioLegend), MyD88 (OTI2B2, ThermoFisher Scientific), TLR7-PE (4G6, Novus Biologicals). Unconjugated antibodies were labeled using the Zip Alexa Fluor 647 Rapid Antibody Labeling Kit (Z11235) as per the manufacturer's instructions (ThermoFisher Scientific). Zombie aqua dye (BioLegend) or live dead fixable green (Thermo Fisher Scientific) was used for detecting dead cells. Cell Fc receptors were blocked using purified rat anti-mouse CD16/CD32 (Mouse BD Fc Block, BD Biosciences) and then stained for 30 min at 4 °C, in the dark, with primary and secondary antibodies. Intracellular staining was performed using the FOXP3 Transcription Factor Staining Buffer Set (eBioscience) according to the manufacturer’s instructions. Samples were acquired on the Fortessa or Fortessa X-20 cytometer with FACSDiva (BD, Biosciences) and analysed using FlowJo v.10 (FlowJo). All fluorescence-activated cell sorting (FACS) and microscopy analysis was carried out at the Microscopy and Cytometry Facility, Australian National University.

### Sanger sequencing

Primers for human *TLR7* DNA sequencing were used at 10 µM (primer sequences available on request). PCR amplification was carried out using Phusion Hot Start II DNA Polymerase II (Thermo Fisher Scientific) and under the conditions recommended by the manufacturer. PCR amplicons were electrophoresed and excised bands were purified using the QIAquick Gel Extraction Kit (Qiagen). Sanger sequencing was completed using Big Dye Terminator Cycle sequencing kit v3.1 (Applied Biosystems) using the same primers used for PCR amplification. Sequencing reactions were run on the 3730 DNA Analyze (Applied Biosystems) system at the ACRF Biomolecular Resource Facility, Australian National University.

### Immunohistochemistry

Liver, pancreas and kidneys were fixed in 10% neutral buffer formalin solution, embedded in paraffin and stained with H&E.

### Bone marrow chimera experimentation

For competitive bone marrow chimeras, *Rag1*^*−/−*^ mice were irradiated and injected intravenously with equal numbers of bone marrow cells from either wild-type or kika CD45.2 and wild-type CD45.1 mice. Mice were given Bactrim in their drinking water for 48 h before injection and for 6 weeks after injection, and housed in sterile cages. After 22 weeks of reconstitution, mice were taken down for phenotyping by flow cytometry.

### B cell culture and Cell Trace Violet staining

Single-cell suspensions were prepared from kika, wild-type or *Tlr7*-knockout mouse spleens. B cells were magnetically purified using the mouse B Cell Isolation Kit (Miltenyi Biotec), labelled with Cell Trace Violet (CTV, Thermo Fisher Scientific) and cultured for 72 h in complete RPMI 1640 medium (Sigma-Aldrich) supplemented with 2 mM l-glutamine (Gibco), 100 U penicillin–streptomycin (Gibco), 0.1 mM non-essential amino acids (Gibco), 100 mM HEPES (Gibco), 55 mM β-mercaptoethanol (Gibco) and 10% FBS (Gibco) at 37 °C in 5% CO_2_. For BCR stimulation, cells were cultured in 10 µg ml^−1^ AffiniPure F(ab′)_2_ fragment goat anti-mouse IgM, µ-chain specific (Jackson Immuno Research) or 1 µg ml^−1^ each R837 (Invitrogen). CD93 expression was examined by sorting splenic B cells with CD19-PE (6D5, BioLegend), CD3-APCCy7 (17A2, BioLegend), CD93-APC (AA4.1, Invitrogen) and the viability stain 7-aminoactinomycin D (Molecular Probes, Invitrogen). Cells were cultured with complete RPMI for 72 h and stimulated with anti-mouse IgM or R837. Bone marrow was obtained from mice. The Fc receptors were blocked (purified rat anti-mouse CD16/CD32 (Mouse BD Fc Block BD Biosciences) and the cells were stained and sorted with B220-PE (RA3-6B2, BioLegend), CD93-APC (AA4.1, Invitrogen) and the viability stain 7-aminoactinomycin D (Molecular Probes, Invitrogen). Cells were sorted on a FACS Aria II system and cultured in complete RPMI medium.

### BMDM cell culture and stimulation

Primary BMDMs from 3 *Tlr7*^*kik/Y*^ mice and wild-type littermates were extracted and differentiated for 7 days in complete DMEM supplemented with L929-conditioned medium as previously reported^47^, before overnight stimulation with ssRNA, guanosine or R848. Noticeably, the yield of *Tlr7*^*kik/Y*^ BMDMs obtained after 7 day differentiation was substantially greater than from wild-type mice. All synthetic RNAs were synthesized by Integrated DNA Technologies. ssRNAs (below) with no backbone modification were resuspended in duplex buffer (100 mM potassium acetate, 30 mM HEPES, pH 7.5, DNase–RNase-free H_2_O), and were previously shown to induce TLR7 sensing in human cells^48^. ssRNAs were transfected with DOTAP (Roche) and pure DMEM in biological triplicate, as previously described^48^, to a final concentration of 500 nM. The ratio of DOTAP to RNA (at 80 μM) was 3.52 µg μl^−1^ of ssRNA. Guanosine (Sigma-Aldrich, G6264, 10 mg freshly resuspended in 176.5 μl DMSO (200 mM stock solution)) and R848 (Invivogen, tlrl-r848) were used at the indicated final concentrations. TNF levels in culture supernatants were detected using the BD OptEIA Mouse ELISA kit (BD Biosciences) according to the manufacturers’ protocols. Tetramethylbenzidine substrate (Thermo Fisher Scientific) was used for quantification of the cytokines on a Fluostar OPTIMA (BMG LABTECH) plate-reader. The RNA sequences used (5′-3′) were as follows: B-406AS-1, UAAUUGGCGUCUGGCCUUCUU; 41-L, GCCGGACAGAAGAGAGACGC; 41-6, GCCGGACAUUAUUUAUACGC; 41-8, GCCGGUCUUUAUUUAUACGC; 41-10, GCCGGUCUUUUUUUUUACGC.

### ADVIA blood analysis

Orbital bleeds were performed on mice and blood samples were run on the ADVIA system (Siemens Advia 1200).

### Western blotting

Cytosolic extracts were prepared from around 20 million–40 million splenocytes by lysis in Triton X-100 buffer (0.5% Triton X-100, 20 mM Tris-HCl pH 7.4, 150 mM NaCl, 1 mM EDTA, 10% glycerol) and centrifuged. Cytosolic extracts were resolved on 8% SDS–polyacrylamide gels and probed with the relevant primary and secondary antibodies. Rabbit anti-TLR7 (D7; Cell Signaling Technology) and mouse anti-mouse TLR7-PE (A94B10; BD Biosciences) were used at 1:1,000, the actin monoclonal antibody (JLA20, Developmental Studies Hybridoma Bank, The University of Iowa) was used at 1:5,000. Membranes were developed with Clarity Western ECL Substrate (BioRad Laboratories).

### Dual-luciferase assays

RAW264.7 cells were transfected with 245 ng of pNIFTY (NF-κB luciferase; InvivoGen), pRL-CMV (100 ng, Promega) *Renilla* luciferase control plasmid, 125 ng of TLR7-HA plasmids (Genecopoeia) expressing the individual variants. After overnight expression, half of the samples were stimulated with 1 mM 2′,3′-cGMP (Santa Cruz) or 1 mM guanosine plus 20 µg ml^−1^ ssRNA using DOTAP for 6 h and dual-luciferase assays were performed as previously described^45^. Raw264.7 cells (originally from ATCC) were tested for mycoplasma contamination using PlasmoTest (InvivoGen).

### Statistics

Statistical analysis was carried out using R software v.3.6.1 (The R Foundation for Statistical Computing) and the Emmeans package. Mouse spleen mass data were analysed using two experiments as a blocking factor and one-way ANOVA, followed by a pairwise estimated marginal means comparison of genotypes. Mouse cellular phenotyping, ELISAs, white blood cell and platelet count analyses were performed using a log linear regression model and one-way ANOVA, followed by a pairwise estimated marginal means comparison of genotypes. Purified B cell cultures were analysed using a linear regression model and one-way ANOVA, followed by a pairwise estimated marginal means comparison of genotypes and stimulatory effect. Luciferase assay statistics were analysed using one-way ANOVA with Bonferroni multiple-comparison test (Prism, GraphPad). All data were filed using Microsoft Excel 2016 and graphed using PRISM.

### DNA, RNA and nRNP ELISAs

Plates were coated with poly-l-lysine (Sigma-Aldrich) before addition of 2.5 µg of either DNA (D7290, Sigma-Aldrich), RNA (AM7120G, Thermo Fisher Scientific) or nRNP (SRC-1000, Immunovision). Plates were then blocked in ELISA blocking buffer (PBS and 1% BSA) for 2 h at room temperature. Mouse serum was diluted 1:40 with ELISA coating buffer (0.05 M sodium carbonate anhydrous/sodium hydrogen carbonate, pH 9.6), and incubated in the ELISA plates overnight at 4 °C. The plates were washed and goat anti-mouse IgG-AP antibodies (Alkaline Phosphatase, Southern Biotech) were added for 1 h at 37 °C. Phosphatase substrate (Sigma-Aldrich, S0942) was used as described by the manufacturer. The samples were read using the Infinite 200 PRO Tecan Microplate Reader (Tecan Group) at an absorbance of 405 nm and normalized to background absorbance at 605 nm.

### Hep-2/*C. luciliae* immunofluorescence

ANAs and dsDNA were determined using Hep-2 and *Crithidia luciliae* slides (both from NOVA Lite), respectively. Serum was diluted 1:40 for Hep-2 slides and 1:20 for *Crithidia* slides and stained as described by the manufacturer using donkey anti-mouse IgG Alexa-488 (Molecular Probes) as the secondary antibody. The slides were imaged using an Olympus IX71 inverted fluorescence microscope.

### RNA-seq analysis

Total B cells were obtained from wild-type or kika mouse spleens and purified using the Mouse B Cell Isolation Kit (Miltenyi Biotec) and stimulated with anti-mouse IgM (10 µg ml^−1^) for 20 h. Total RNA was extracted using RNeasy Mini Kits (74104, Qiagen). Sequencing was performed using the NextSeq500 platform and analysis was conducted using the following R packages: limma, edgeR and enhanced volcano^49^. For the patient, type I IFN single-cell RNA-seq analysis was performed. PBMCs were isolated from frozen human samples as previously described^50^. Live cells were next purified by FACS using 7AAD and labelled with TotalSeq anti-human hashtags (BioLegend). The number of cells was determined and 10,000 cells per sample were run on the 10x Chromium platform (10x Genomics). Library preparation and sequencing were performed by The Biomedical Research Facility according to the manufacturer’s instructions for the Chromium Next GEM Single Cell 5′ Kit v2. The samples were sequenced using the NovaSeq 6000 (Illumina) system. The FASTQ files were aligned to the human GRCh38 reference genome using 10x Genomics Cell Ranger pipeline v.6.0.1. Statistical analysis, clustering and visualization were conducted using Seurat v.4.0.1 in the R environment.

### Molecular dynamics simulations

Details of the computational modelling are provided in the Supplementary Methods.

### Reporting summary

Further information on research design is available in the Nature Research Reporting Summary linked to this paper.

## Online content

Any methods, additional references, Nature Research reporting summaries, source data, extended data, supplementary information, acknowledgements, peer review information; details of author contributions and competing interests; and statements of data and code availability are available at 10.1038/s41586-022-04642-z.

## Supplementary information

Supplementary Information Supplementary Tables 1-4 and Supplementary Methods, containing details about the computational modelling.

Reporting Summary

## Data Availability

For the genomic data relating to the families in Fig. 1, family A has been deposited in SRA, under BioProject accession number PRJNA798834; family B has been deposited at the Baylor-Hopkins Center for Mendelian Genomics, AnVIL repository under the participant IDs BH14453-1 (proband), BH14453-2 (mother) and BH14453-3 (father); family C has been submitted to the EGA (EGAS00001005965). Sequencing data in Fig. 3i have been deposited at the Gene Expression Omnibus under accession number GSE196316 and Fig.4o has been deposited at Open Science Framework (OFS) under accession number pw62n.
