# Supplementary material for: TLR7 gain-of-function genetic variation causes human lupus
Source: Nature. 2022 Apr 27;605(7909):349–56. doi: 10.1038/s41586-022-04642-z (PMC9095492; doi:10.1038/s41586-022-04642-z)
Supplement: Supplementary file 1 — Supplementary Tables 1-4 and Supplementary Methods, containing details about the computational modelling. [file 41586_2022_4642_MOESM1_ESM.pdf]

nature portfolio

https://doi.org/10.1038/s41586-022-04642-z

Supplementary information

TLR7 gain-of-function genetic variation causes human lupus

In the format provided by the authors and unedited

Supplementary Materials for

TLR7 gain-of-function genetic variation causes human lupus

Grant J. Brown[1], Pablo F. Cañete*[1], Hao Wang*[1], Arti Medhavy[1], Josiah Bones[2], Jonathan A. Roco[1], Yuke He[3], Yuting Qin[3,] Jean Cappello[1], Julia I. Ellyard[1], Katharine Bassett[1], Qian Shen[1], Gaetan Burgio[1], Yaoyuan Zhang[1], Cynthia Turnbull[1], Xiangpeng Meng[1], Phil Wu[1], Eun Cho[1], Lisa Miosge[1], T. Daniel Andrews[1], Matt A. Field[1,4], Denis Tvorogov[5], Angel F. Lopez[5], Jeffrey J. Babon[6], Cristina Aparicio López[7], África Gónzalez-Murillo[8, 9], Daniel Clemente Garulo[10], Virginia Pascual[11], Tess Levy[12,13] Eric J. Mallack[14], Daniel G. Calame[15, 16, 17], Timothy Lotze[15, 16], James R. Lupski[16, 17, 18, 19], Huihua Ding[3, 20], Tomalika R. Ullah[21, 22], Giles D. Walters[23], Mark E. Koina[24], Matthew C. Cook[1], Nan Shen[3, 20, 25], Carmen de Lucas Collantes[7, 26], Ben Corry[2], Michael P. Gantier[20, 21], Vicki Athanasopoulos[1#] and Carola G. Vinuesa[1, 5, 27#].

# and * = Equal contribution

Correspondence to: *carola.vinuesa@anu.edu.au*, carola.vinuesa@crick.ac.uk

This includes:

Supplementary Tables 1 to 4

Supplementary Method for Computational modeling

Supplementary Tables

ID	Sex	Age of onset	Ethnicity	Diagnosis	Clinical and laboratory manifestations
A.II.1	F	7	European /Caucasian	SLE with CNS and renal involvement	Presented with refractory immune thrombocytopenia and hemichorea. Elevated ANAs (EIA: 763, normal range <1; anti-DNA IFI positive > 1/1280, homogeneous nuclear pattern) and hypocomplementemia (C3 37.9 mg/dl, normal range: 76 – 184; C4: 5.75 mg/dl, normal range: 16 – 47). Antibodies to extractable nuclear antigens were negative. She also suffered from inflammatory arthralgias and constitutional symptoms. Renal involvement a year later when she presented with a hypertensive crisis (140-150 / 80 mmHg > P99 for her height and sex). Blood pressure control required treatment with enalapril, amlodipine and spirinolactone, adjusting the doses periodically. Renal biopsy has not been performed. She also has mild mitral insufficiency. Hematologically, she has presented severe thrombocytopenia (minimun 8.000/μL) that has required treatment with prednisone, azathioprine, mycophenolate, rituximab, immunoglobulins and Etanercept. Has suffered intermittent episodes of chorea, treated with haloperidol.
B.II.1	F	9	American/ Caucasian	Neuromyelitis optica	Presented at age 9 with relapsing optic neuritis and transverse myelitis. Found to have NMO/AQP4-IgG in serum and CSF. ANA profile demonstrated positive ANA (1:320, speckled) without other autoantibodies. First treated with rituximab but had additional NMO exacerbations leading to treatment with IVIG and mycophenolate mofetil. Developed neutralizing antibodies to rituximab and early B cell repopulation, resulting in discontinuation of rituximab and recent initiation of inebilizumab-cdon. Mother has hemiplegic cerebral palsy of unclear etiology and developed SLE in mid-20s.
C.I.1	F	18	Chinese/ East Asian	SLE	Presented with malar rash, joint pain, Raynaud's phenomenon, alopecia, fever and oral ulcers. At the time of sample collection, she was readmitted to the hospital due to recurrent fever, headache, anxiety, and significantly decreased WBC. Auto-ab panel revealed positive ANA (1:1280), U1RNP, SSA-Ro52, SSA-Ro60, and dsDNA (Farr 42.55U/ml). FBC showed lymphocytopenia (0.26 * 10^9/ml), thrombocytopenia (55 * 10^9/ml). CM-CSF corrected the WBC. She also had low C3 [0.785g/L (0.9-1.8)]. CNS evaluation could not be performed. Active episode treated with methylprednisolone 80mg IV.

Supplementary Table 1. Ethnicity, diagnosis and clinical manifestations in probands carrying *TLR7* variants

Gene	*Base change*	*Amino acid substitution*	*SIFT*	*Polyphen-2*	*CADD*	*gnomAD*	*ExAC (%)*
TLR7	c.790T>C	p.Tyr264His	0.12	0.443	22.5	N/A	N/A
TLR7	c.82A>G	p.Arg28Gly	0.35	0.05	12.79	N/A	N/A
TLR7	c.1521T>G	p.Phe507Leu	0.00	0.874	22.4	N/A	N/A

Supplementary Table 2. ***TLR7*** **variants, frequency and its predicted damage using *in silico* tools.** CADD = Combined Annotation Dependent Depletion; gnomAD = The Genome Aggregation Database; ExAC = Exome Aggregation Consortium.

Gene	Variants
C1QA	
C1QB	
C1QC	
C1R	
C2,CFB	
C4A	
C4B	
DNASE1	
TREX1	
PRKCD	
DNASE1L3	
ACP5	
TNFSF6	
IFIH1	
SOCS1	
NCKAP1L	
C1S	
C3	
SAMHD1	
ADAR1	
**RNASEH2B**	p.Ala177Thr
TMEM173	

Supplementary Table 3. Rare variants of genes causing human SLE.

Organs / Mice	Liver	Skin	Brain	Lung	Heart

**+/Y (n=3)**	midzonal congestion and acute multifocal hepatocellular necrosis(n=1)	NAD	NAD	NAD	cardiomyocyte degeneration and hypertrophy(n=2)
***kik*/Y (n=3) and *kik*/*kik* (n=3)**	probable histiocytic sarcoma metastases(n=1)<br><br>lymphocytic infiltrate(n=3)<br><br>chronic active cholangiohepatitis(n=1)<br><br>extramedullary haematopoiesis(n=1)	panniculus muscle focal myocyte degeneration and necrosis(n=1)<br><br>myocyte degeneration and attempted regeneration(n=1)	NAD	focal sub pleural lymphocytic infiltrate(n=1)<br><br>chronic lymphadenitis(n=3)<br><br>focal atelectasis(n=1)<br><br>sub pleural and perivascular lymphocytic/blastic infiltration(n=1)<br><br>mild focal interstitial mononuclear cell infiltration(n=1)	focal myocardial fibrosis(n=2) and mucoid degeneration(n=1)<br><br>cardiomyocyte degeneration and hypertrophy(n=1)

Organs / Mice	**Stomach**	**Gall bladder**	**Urinary Bladder**	**Thymus**	**Salivary glands Regional lymph nodes**	**Pancreas**
**+/Y (n=3)**	submucosal eosinophil and fibrohistiocytic infiltration (n=1)	NAD	NAD	NAD	Lymphocytosis(n=1)	NAD
***kik*/Y (n=3) and *kik*/*kik* (n=3)**	individual glandular dilation with attenuation of lining epithelium(n=1)<br><br>Submucosal mononuclear cell/eosinophil infiltration and few dilated glands containing eosinophilic crystalline shards(n=1)	NAD	chronic arteritis /periarteritis with mural fibrinoid necrosis(n=1)	small in size with loss of clear corticomedullary demarcation, paucity of typical cortical cells(n=1)	chronic lymphadenitis(n=4)	lymphocytic and fatty infiltration with glandular atrophy(n=1)<br><br>chronic lymphadenitis(n=1)<br><br>focal acute pancreatitis(n=1)

Organs / Mice	**Spleen**	**Mesenteric lymph node & inguinal lymph node**	**Cecum Colon GALT**	**Small intestine (Duodenum, Jejunum, Ileum)**
**+/Y (n=3)**	12x5x3mm;<br>12x5x3mm;<br>15x5x3mm;<br>splenic congestion(n=1)	NAD	NAD	focal mural macrophage and eosinophil infiltration(n=1)
***kik*/Y (n=3) and *kik*/*kik* (n=3)**	18x6x3mm;<br>27x18x4mm, large;<br>17x6x3mm;<br>16x6x4mm;<br>20x6x3mm, large;<br>30x10x7mm, large<br><br>early lymphoma (n=2) and lymphoma(n=2)<br><br>splenic arteriolar fibrinoid necrosis(n=1)<br><br>chronic lymphadenitis(n=1)congested with paucity of mature lymphocytes(n=1)	chronic lymphadenitis(n=6)	lymphoid hyperplasia(n=1)<br><br>chronic mesenteric lymphadenitis(n=1)<br><br>lymphoid hyperplasia in Peyer's patches(n=1)	focal mucosal inflammation plus single dilated gland with enterocyte apoptotic bodies in lumen(n=1)<br><br>villous lymphatic dilation(n=1)<br><br>chronic enteritis and mesenteric lymphadenitis(n=1)<br><br>reactive changes within normal limits(n=1). Lymphoid hyperplasia in Peyer's Patches(n=3)

Supplementary Table 4. Histopathology observations. NAD= no abnormalities detected.

Supplementary Method for Computational Modeling

Starting coordinates: To compare binding affinities of guanosine monophosphate and R848 in the WT and Y264H TLR7 we used thermodynamic integration [1] in AMBER20/AMBERTOOLS21 [2] to calculate the energetic cost to remove each bound ligand. To account for the uncertain protonation state of histidine we used 3 protein systems: Y264, Y264H with a neutral epsilon protonated histidine, and Y264H$^+$ with a charged double protonated histidine. The protein structure (PDB: 6IF5 [3]) was mutated from the original monkey sequence using CharmmGUI [4-6]. Glycan cores present in the crystal structure were kept for the simulation. The ligands from this structure were removed and guanosine and R848 were added using geometries extracted from analogous PDB files (5GMF, 5GMH) [7] after alignment of the protein backbone of each structure around the binding site. We noted the geometries of all utilised structures had excellent consistency. Protonation states for histidine residues were selected based on calculations using PROPKA [8]. For the *kika* mutant, these calculations suggested it may be neutral or charged at physiological pH, explaining our choice to test both variants explicitly.

Force field parameters: Bonded force field parameters for each ligand were generated by analogy to the GAFF2 parameter set through the use of AMBER's antechamber and parmcheck programs. RESP charge parameters were then calculated in GAUSSIAN09 [9] with the help of FFTK in VMD [10], using the geometries from the aforementioned crystal structures. AMBER ff19SB [11], OPC [12], and GLYCAM-06 [13] were used for protein, water, and glycan parameters accordingly. GLYCAM-06 was modified for the connecting protein residues to update them with ff19SB bonded parameter and CMAP corrections for improved consistency with the rest of the system. For ion parameters, the 12-6 IOD OPC set was used from Sengupta et al [14]. Thermodynamic integration in AMBER20 currently does not support 12-6-4 ion parameters, explaining the omission of these improved parameters in this study.

System building: System building was executed in tleap, where each prepared system was solvated with a distance of 10Å to the system box edge. 150 mM NaCl was then added by random replacement of water molecules. The ion count corresponding to this concentration was calculated by two iterations of SLTCAP [15] on the Y264 system: In the first, we input sum protein and glycan mass as determined by an initial non-solvated tleap run, desired salt concentration, protein net charge, and the corresponding number of water molecules as determined by initial solvation with tleap. In the second, we updated the number of waters, accounting for the removal of some water from the first pass due to the ion addition. This final ion count was used for all systems, except for the versions of the system where the dimer was mutated to Y264H$^+$, where we added an extra two chloride ions to neutralise them. The resulting built systems contained around 208800 atoms, including all contributions by the four-point water model. After building, systems were post-processed with parmed [16] for hydrogen mass repartitioning to facilitate a 4 fs timestep.

Simulation parameters: Harmonic position restraints with a force constant of 5 kcal/mol/Å^2 were applied to CA, C and N backbone atoms of the protein that were greater than approximately 8Å from binding site in order to assist in convergence. Any region making up the contact between both protomers was not included in this definition, and some additional regions beyond corresponding to locations with substantial sequence difference to the original monkey sequence, or terminal residues before the two unresolved loops. The same restraints were also applied to heavy atoms in the glycans to avoid large changes from their crystal geometry. The non-terminal resolved Z-Loop region was gently restrained with a force constant of 0.1 kcal/mol/Å^2 on the backbone atoms. To assist in convergence of thermodynamic integration, and to remove the need for translational sampling of the ligand, 6 degree-of-

freedom ligand-protein restraints were prepared according to criteria covered by Boresch et al. [17]. For these Boresch restraints, the single distance restraint was held with a force constant of 25 kcal/mol/Å^2, while the two angle, and three dihedral restraints were held with a force constant of 100 kcal/mol/radian^2. Additional intra-ligand dihedral restraints were also prepared to make the ligand essentially rigid during electrostatic removal and ligand decoupling. This was achieved using modification of the BAT.py code by Heinzelmann and Gilson [18]; all non-hydrogen dihedrals were restrained with a weight of 5 kcal/mol/radian^2. Both the Boresch and intra-ligand dihedral restraints were applied to both ligands in the dimer system, although only one ligand would undergo thermodynamic integration. **The geometric details of the Boresch restraints, and the residues stabilised by the 5 kcal/mol/Å^2 harmonic restraints can be found in the supplementary material.** System temperature was controlled under a Langevin thermostat with a collision frequency of 2 ps^-1 and a target temperature of 298.15 K. Pressurisation utilised a Monte Carlo barostat with isotropic position scaling and a pressure relaxation time of 0.5 ps, to a target pressure of 1 bar.

System equilibration: During heating and pressurisation, all other backbone atoms not previously restrained in the system were harmonically positionally restrained with a force constant of 1 kcal/mol/Å^2. Before thermodynamic integration commenced, all replicas of all systems were first minimised without SHAKE for 10000 steps of steepest decent with CPU code. A further 1000 steps followed with SHAKE turned on, which was then used for all simulations after this point. All later simulations were executed on GPU code. To equilibrate to temperature and pressure targets on GPU code, five restarting simulation legs were run for 0.1 ns each at a timestep of 1 fs in order to avoid large changes in simulation volume within a single simulation, which would otherwise likely result in crashes. The temperature was linearly increased from 0 K to 298.15 K through these five simulations under pressure. From here, all later simulations were run at a 4 fs timestep. To ensure pressure was stable after the initial combined heating and pressurisation simulations, a further 2.5 ns of pressurisation was carried out, restarting every 0.5 ns. After this, the equilibration restraints were eliminated in linearly reducing restarting increments, from 1 to 0.1 kcal/mol/Å^2 over 10 simulations of 0.25 ns length each, and from 0.1 to 0 kcal/mol/Å^2 over 10 simulations of 0.25 ns length each. Each system was then run for a further 5 ns to ensure stability. The lambda value used during equilibration was always the value corresponding to the most restrained, charged, or coupled state of the ligand for a given leg of the thermodynamic cycle. The coordinates at the end of equilibration were utilised as the starting point for all lambda windows of a given leg and replica, albeit with new randomly generated velocities.

Thermodynamic integration: Relative binding free energies of the ligands to each protein variant were determined using the method of thermodynamic integration in which the ligand is progressively decoupled from the protein during a series of simulations. This was done by breaking the process into a series of legs that describe the energy to remove the ligand from the protein. These included the cost to turn on the 6 degree-of-freedom and intra-ligand dihedral restraints explicitly ($\Delta G1$), the cost to remove electrostatic interactions with the surrounding protein environment ($\Delta G2$), and the cost to decouple the van der Waals interaction of the ligand with the protein ($\Delta G3$). $\Delta G3$ was calculated with the ligand defined as softcore under AMBER20's second order smoothstep function [19] , which ensures endpoint contributions to the free energy are 0. We utilised an alpha value of 0.5 during the softcore phase and scaled intra-ligand electrostatics with lambda to avoid spurious gas phase self-interactions. Restraints were not scaled with lambda, except for when their contribution was being calculated explicitly. We finish by analytically accounting for the 6DoF ligand-protein restraints as described by Boresch et al. [17] ($-\Delta G4$). The relative binding free energy between systems of a

shared ligand is determined from the sum of these components for each mutant relative to the WT value. Three replicates of each leg of the thermodynamic cycle were prepared with their own equilibration phase as detailed earlier. Each lambda window for a given replica was then split from the equilibration phase with new random starting velocities, and further equilibrated for 1 ns. Data from equilibration would not be included in analysis. For production runs, data was collected every 1 ps. 21 lambda windows per replica were used to calculate ΔG1, 25 for ΔG2, and 48 for ΔG3. Each window used to calculate ΔG1 was run for 10 ns per window, while those used for calculating ΔG2 and ΔG3 were run for 50 ns in the case of guanosine systems, and 20 ns in the case of R848 systems. To conserve computational resources, one replica for the calculation of guanosine's ΔG3 value had some windows run for less time when they were considered to have already converged with a within-window standard error < 0.25 kcal/mol, resulting in windows of varying runtime from 10 to 50 ns. The total production runtime of all systems, replicates, and windows summed to 45.390 μs.

Analysis: Thermodynamic integration analysis was conducted using alchemlyb [20] and pymbar [21]. Data from production regions were subsampled using pymbar's conservative algorithm to ensure data points were decorrelated to acquire accurate error estimates. Data fitting was executed once for each system and leg of the thermodynamic cycle by pooling decorrelated data for each replica by window. The fit was executed using alchemlyb. The final reported value would include a summation of all three simulation legs (ΔG1+ΔG2+ΔG3) along with the analytical calculation (-ΔG4) and the accumulative standard error. To analyse the presence of water near residue 264, VMD [10] was used to count the number of water molecules within 3.5 Å of all hydrogen and oxygen atoms belonging to the tail regions of the ligands. **Details of atoms included in this definition are in Extended Data Fig. 1e**. The trajectory frames used for this calculation were sourced from windows with lambda 0.0001 of the replicas calculating ΔG2, which were pooled by system with n=150 for each guanosine system, and n=60 for each R848 system. These windows correspond to a near-fully coupled state of the ligand. The final values report the average number of waters bound to the sterically hindered tail of the ligand by mutation, with standard error. **Figure 1h-i and Extended Data Fig. 1e-f** was prepared in pymol [22] using the trajectory frame taken after 10 ns of production thermodynamic integration from replica 1 of guanosine runs of Y264 and Y264H$^+$, ΔG2, lambda 0.0250.

1 Lee, T. S. *et al.* Alchemical binding free energy calculations in AMBER20: Advances and best practices for drug discovery. *Journal of Chemical Information and Modeling* **60**, 5595-5623, doi:10.1021/acs.jcim.0c00613 (2020).

2 AMBER 2021 Reference Manual (2021) https://ambermd.org/doc12/Amber21.pdf.

3 Zhang, Z., Ohto, U. & Shimizu, T. Crystal structure of monkey TLR7 in complex with 2',3'-cGMP (Guanosine 2',3'-cyclic phosphate). doi:10.2210/pdb6IF5/pdb (2018).

4 Jo, S., Kim, T., Iyer, V. G. & Im, W. CHARMM-GUI: A web-based graphical user interface for CHARMM. *Journal of Computational Chemistry* **29**, 1859-1865, doi:10.1002/JCC.20945 (2008).

5 Yuan, M. *et al.* A highly conserved cryptic epitope in the receptor binding domains of SARS-CoV-2 and SARS-CoV. *Science (New York, N.Y.)* **368**, 630-633, doi:10.1126/science.abb7269 (2020).

6 Koo, J. R. *et al.* Interventions to mitigate early spread of SARS-CoV-2 in Singapore: a modelling study. *The Lancet. Infectious diseases* **20**, 678-688, doi:10.1016/s1473-3099(20)30162-6 (2020).

7 Zhang, Z. *et al.* Structural Analyses of Toll-like Receptor 7 Reveal Detailed RNA Sequence Specificity and Recognition Mechanism of Agonistic Ligands. *Cell Reports* **25**, 3371-3381.e3375, doi:10.1016/j.celrep.2018.11.081 (2018).

8 Søndergaard, C. R., Olsson, M. H., Rostkowski, M. & Jensen, J. H. Improved Treatment of Ligands and Coupling Effects in Empirical Calculation and Rationalization of pKa Values. *J Chem Theory Comput* **7**, 2284-2295, doi:10.1021/ct200133y (2011).

9 Gaussian 09, Revision D.01 (Gaussian, Inc., Wallingford CT, 2016).

10 Humphrey, W., Dalke, A. & Schulten, K. VMD: Visual molecular dynamics. *Journal of Molecular Graphics* **14**, 33-38, doi:10.1016/0263-7855(96)00018-5 (1996).

11 Tian, C. *et al.* Ff19SB: Amino-Acid-Specific Protein Backbone Parameters Trained against Quantum Mechanics Energy Surfaces in Solution. *Journal of Chemical Theory and Computation* **16**, 528-552, doi:10.1021/acs.jctc.9b00591 (2020).

12 Izadi, S., Anandakrishnan, R. & Onufriev, A. V. Building water models: A different approach. *Journal of Physical Chemistry Letters* **5**, 3863-3871, doi:10.1021/jz501780a (2014).

13 Kirschner, K. N. *et al.* GLYCAM06: A generalizable biomolecular force field. Carbohydrates. *Journal of Computational Chemistry* **29**, 622-655, doi:10.1002/JCC.20820 (2008).

14 Sengupta, A., Li, Z., Song, L. F., Li, P. & Kenneth M. Merz, Jr. Parameterization of Monovalent Ions for the OPC3, OPC, TIP3P-FB, and TIP4P-FB Water Models. *Journal of Chemical Information and Modeling* **61**, 869-880, doi:10.1021/ACS.JCIM.0C01390 (2021).

15 Schmit, J. D., Kariyawasam, N. L., Needham, V. & Smith, P. E. SLTCAP: A Simple Method for Calculating the Number of Ions Needed for MD Simulation. *Journal of Chemical Theory and Computation* **14**, 1823-1827 (2018).

16 Shirts, M. R. *et al.* Lessons learned from comparing molecular dynamics engines on the SAMPL5 dataset. *Journal of Computer-Aided Molecular Design 2016 31:1* **31**, 147-161, doi:10.1007/S10822-016-9977-1 (2016).

17 Boresch, S., Tettinger, F., Leitgeb, M. & Karplus, M. Absolute binding free energies: A quantitative approach for their calculation. *Journal of Physical Chemistry B* **107**, 9535-9551, doi:10.1021/jp0217839 (2003).

18 Heinzelmann, G. & Gilson, M. K. Automation of absolute protein-ligand binding free energy calculations for docking refinement and compound evaluation. *Scientific reports* **11**, 1116-1116, doi:10.1038/s41598-020-80769-1 (2021).

19 Lee, T. S. *et al.* Improved Alchemical Free Energy Calculations with Optimized Smoothstep Softcore Potentials. *Journal of Chemical Theory and Computation* **16**, 5512-5525, doi:10.1021/acs.jctc.0c00237 (2020).

20 alchemlyb: the simple alchemistry library version 0.3.1 (2021).

21 Shirts, M. R. & Chodera, J. D. Statistically optimal analysis of samples from multiple equilibrium states. *The Journal of Chemical Physics* **129**, 124105-124105, doi:10.1063/1.2978177 (2008).

22 The PyMOL Molecular Graphics System, Version 2.5.1 (2021).
