# Supplementary material for: TLR7 gain-of-function genetic variation causes human lupus
Source: Nature. 2022 Apr 27;605(7909):349–56. doi: 10.1038/s41586-022-04642-z (PMC9095492; doi:10.1038/s41586-022-04642-z)
Supplement: Supplementary file 2 — Reporting Summary [file 41586_2022_4642_MOESM2_ESM.pdf]

Corresponding author(s): Carola Vinuesa
Last updated by author(s): Jan 12, 2022

Reporting Summary

Nature Research wishes to improve the reproducibility of the work that we publish. This form provides structure for consistency and transparency in reporting. For further information on Nature Research policies, see our Editorial Policies and the Editorial Policy Checklist.

Statistics

For all statistical analyses, confirm that the following items are present in the figure legend, table legend, main text, or Methods section.

n/a	Confirmed	
☐	☒	The exact sample size (*n*) for each experimental group/condition, given as a discrete number and unit of measurement
☐	☒	A statement on whether measurements were taken from distinct samples or whether the same sample was measured repeatedly
☐	☒	The statistical test(s) used AND whether they are one- or two-sided<br>*Only common tests should be described solely by name; describe more complex techniques in the Methods section.*
☐	☒	A description of all covariates tested
☐	☒	A description of any assumptions or corrections, such as tests of normality and adjustment for multiple comparisons
☐	☒	A full description of the statistical parameters including central tendency (e.g. means) or other basic estimates (e.g. regression coefficient) AND variation (e.g. standard deviation) or associated estimates of uncertainty (e.g. confidence intervals)
☐	☒	For null hypothesis testing, the test statistic (e.g. *F*, *t*, *r*) with confidence intervals, effect sizes, degrees of freedom and *P* value noted<br>*Give P values as exact values whenever suitable.*
☒	☐	For Bayesian analysis, information on the choice of priors and Markov chain Monte Carlo settings
☒	☐	For hierarchical and complex designs, identification of the appropriate level for tests and full reporting of outcomes
☒	☐	Estimates of effect sizes (e.g. Cohen's *d*, Pearson's *r*), indicating how they were calculated

Our web collection on statistics for biologists contains articles on many of the points above.

Software and code

Policy information about availability of computer code

Data collection	Cellular phenotype data was collected on a Fortessa or Fortessa X-20 cytometer running FACSDiva version 8.0 (BD, Biosciences).
Data analysis	Statistical analysis was carried out using R software version 3.6.1 (The R Foundation for Statistical Computing) and the Emmeans package. Luciferase assays statistics were analysed using a one-way ANOVA with Tukey's multiple comparison (PRISM 6, GraphPad Software LLC). Flow cytometry data was analysed using the FlowJo software v10 (FlowJo LLC). Microsoft Excel 2016, Sequencher v5. Single-cell data was analysed using Cell Ranger v6.0.1 pipeline.

For manuscripts utilizing custom algorithms or software that are central to the research but not yet described in published literature, software must be made available to editors and reviewers. We strongly encourage code deposition in a community repository (e.g. GitHub). See the Nature Research guidelines for submitting code & software for further information.

Data

Policy information about availability of data

All manuscripts must include a data availability statement. This statement should provide the following information, where applicable:
- Accession codes, unique identifiers, or web links for publicly available datasets
- A list of figures that have associated raw data
- A description of any restrictions on data availability

The authors declare that data supporting the findings of this study are available within the paper or its supplementary information files. Data that support the findings of this study are also available from the corresponding author (CGV) upon reasonable request.

Field-specific reporting

Please select the one below that is the best fit for your research. If you are not sure, read the appropriate sections before making your selection.

☒ Life sciences ☐ Behavioural & social sciences ☐ Ecological, evolutionary & environmental sciences

For a reference copy of the document with all sections, see nature.com/documents/nr-reporting-summary-flat.pdf

Life sciences study design

All studies must disclose on these points even when the disclosure is negative.

Sample size	For each experiment we estimated the expected change between experimental and control groups (e.g. at least a 20% change and SD at most half the magnitude of the minimum effect size we were interested in). With those assumptions we used power analysis to estimate the group size that would provide at least 80% power to detect statistically-significant difference (with $p < 0.05$ considered significant).
Data exclusions	No data was excluded
Replication	Figs 1a-d, g-i, 1k (survival curve) no experimental replication is feasible.<br>NF-κB luciferase assays in Fig 1e, f is representative of 2 experiments.<br>Fig 1j results are representative of 4 experiments for spleen mass and 4 pooled experiments for cellularity, 3 for serum ELISAs (Fig 1m), 1 times for ANAs (Fig 1l).<br>Figure 1n (R848) used 2 mice per genotype (done in triplicate wells) and done on the same day. Fig 1o (Guanosine) used 3 mice per genotype (all in triplicate wells). Done on two different days.<br>Platelet analysis (fig 2a) was done once and the organ H&E analysis and electron microscopy (fig 2b-c) 3 times. H&E stain in fig 2d was done once, and serum level in Fig 2e were assayed once using large numbers of mice. Figs. 2f-h,j-l of the splenic phenotyping in Figs 2f-h,j-l are representative of four experiments whilst the kidney phenotyping analysis (Fig2i) is representative of two experiments.<br>Fig 3a-b were done once. Fig 3g, the B cell culture was done 3 times.<br>Fig3c-d: representative of two experiments, and Fig3h is a compilation of two experiments. No experimental replication was feasible for Fig 3i. Western blotting results in Fig 3e-f are representative of 3 experiments each.<br>Figs 4a-e, the MyD88-/- crosses were done once. Figs. 4f-h are representative of two experiments and the Western blots in Fig 4i were replicated at least 2 times for each antibody. Figs. 4j-o: no experimental replication was feasible.<br>FigS1a-f no experimental replication feasible.<br>FigS2a no experimental replication feasible. FigS2b was done once.<br>Fig S3a, b, c was done once.<br>FigS3d-f is representative of 1 experiment. FigS3 g (dose response) used 2 mice per genotype (all in triplicate wells). Done on the same day.<br>Fig S3h (ssRNA): used 3 mice per genotype (all in triplicate wells). Done on two different days.<br>Fig S4 was carried out once on organs from 3 mice of each genotype.<br>FigS5a ADVIA blood analysis and analyses of chimeras in FigS5h-j were done once, blood flow cytometry twice (Fig S5c, e) and splenic flow four times for FigS5b, f, and g. Fig S5k is a compilation of 2 experiments.<br>FigS6b-g results are representative of 2 experiments. FigS6h is a compilation of five litters of genotyping results. Fig S6i was done twice.<br>FigS7a, c, d is representative of 1 experiment, whilst 4 splenic B cell cultures were used in Fig S7b.<br>FigS8a was done once.<br>FigS9 was done once.
Randomization	For in vitro experiments, randomisation was not required given there were no relevant covariates (i.e. cells from littermate mice came from the same cage, all wells treated simultaneously using multi-channel pipettes, on the same day, in the same single plate, analysed in the same machine, handled by the same investigator).
Blinding	Blinding to allocation occurred for all experiments in which the investigator had to score data manually (i.e. intensity and pattern of ANA fluorescence, analysis of histological samples from mouse necropsies, assessment of Ig deposits in EM kidney sections). Blinding did not occur for assays in which a pre-determined order was required for loading gels plus the result would be presented raw to the reader (i.e. western blot gels) or analysed via an automated machine without input from the investigator (i.e. quantification of luciferase activity). Investigators planned mouse experiments based on genotype and grouping, but during performance of experiments mice were identified only by randomly assigned number with investigators blind to group allocation.

Reporting for specific materials, systems and methods

We require information from authors about some types of materials, experimental systems and methods used in many studies. Here, indicate whether each material, system or method listed is relevant to your study. If you are not sure if a list item applies to your research, read the appropriate section before selecting a response.

Materials & experimental systems

n/a	Involved in the study
☐	☒ Antibodies
☐	☒ Eukaryotic cell lines
☒	☐ Palaeontology and archaeology
☐	☒ Animals and other organisms
☐	☒ Human research participants
☒	☐ Clinical data
☒	☐ Dual use research of concern

Methods

n/a	Involved in the study
☒	☐ ChIP-seq
☐	☒ Flow cytometry
☒	☐ MRI-based neuroimaging

Antibodies

Antibodies used

All antibodies used are commercially availiable and extensively used. We have listed all antibodies and their clone names in the materials section but given the large number of antibodies used over the breadth of the work we did not note all their lot numbers. SiglecH-APC (#551, Biolegend), IgD-FITC (#405718, Biolegend), IgD-PerCP Cy5.5 (#11-26c.2a, BD Pharmingen), CD3-A700 (#17A2, BioLegend), CD19- BUV395 (#1D3, BD Horizon), CD138-PE (#281-2, BD Pharmingen), PD1-BV421 (#29F.1A12, BioLegend), CCR7-PerCP Cy5.5 (#4B12, BioLegend), CD8-BUV805 (#53-6.7, BD Horizon), CD19-BV510 (#6D5, BioLegend), CD4-BUV395 (#6K1.5, BD Horizon) CD21/35-BV605 (#7G6, BD Horizon), CD45.1-BV605 (#A20, BioLegend), CD45.1-BV711 (#A20, BioLegend), CD45.1-PB (#A20, BioLegend, TLR7-PE (#A94B10, BD Pharmingen), CD23-BV421 (#B3B4, BioLegend), CXCR3-PE (#CXCR3-173, BioLegend), CD19-A700 (#eBio1D3, Invitrogen), FoxP3-FITC (#FJK-16s, Invitrogen (eBioscience), FoxP3-PECy7 (#FJK-16s, Invitrogen eBioscience), IgM-FITC (#II/41, BD Pharmingen), IgM-PECy7 (#II/41, Invitrogen), CD44-FITC (#IM7, BD Pharmingen), CD44-PB (#IM7, BioLegend), CD95 (FAS)-BV510 (#Jo2, BD Horizon), BCL6-A467 (#K112-91, BD Pharmingen), CD11b-PerCP Cy5.5 (#M1/70, BioLegend), IA/IE-BV421 (#M5/114.15.2, BioLegend), CD11c-A647 (#N418, BioLegend), CD11c-BV510 (#N418, BioLegend), CD11c-FITC (#N418, BioLegend), CD25-PE (#PC62, BioLegend), B220-A647 (#RA3-6B2, BD Pharmingen), B220-BUV395 (#RA3-6B2, BD Horizon), B220-BUV737 (#RA3-6B2, BD Horizon), CD98-PECy7 (#RI.388, BioLegend), CD4-PECy7 (#RM4-5, BD Pharmingen), CD25-A647 (#PC61, Biolegend), CD4-A647 (#RM4-5, Biolegend), CD11c-APC (#HL3, BD Pharmingen), CD138-Biotin (#281-2, BD Bioscience), CXCR5-Biotin (#2G8, BD Bioscience), Streptavidin-BUV805 (BD Horizon), Streptavidin-BV510 (BioLegend), CD19-BV605 (#6D5, Biolegend), B220-PE (#RA3-6B2, Biolegend), BST2-PE (#927, Biolegend), CD19-PE (#6D5, Biolegend), IgD-PE (#11-26c.2a, Biolegend), CD11b-PECy7 (#M1/70, eBiosciences), Streptavidin-PECy (eBiosciences), CD4-PerCPCy5.5 (#RM4-5, Biolegend), CD45.2-PerCPCy5.5 (#104, BD Bioscience), CD3-Pacific Blue (#HIT2, BD pharmingen). For human PBMCs: CD19-BV650 (#HIB19, Biolegend), HLA-DR-BV510 (#L243, Biolegend), CD 24-BV605 (#ML5, Biolegend), CD56-PECy7 (#NCAM16.2, BD pharmingen), CD14-PerCP (#MΦP9, BD pharmingen), IgD-BV510 (#IA6-2, Biolegend), CD123-PE (#7G3, BD pharmingen), CD21-APC (#B-ly4, BD pharmingen), CD11c-APC (#B-ly6, BD pharmingen), CD16-APC-H7 (#3G8, BD pharmingen), IgG-PECy7 (#G18-145, BD pharmingen), CD10-PE-CF594 (#HI10a, BD pharmingen), IgA-PE (#IS11-8E10, Miltenyi Biotech), CD27-APC-EF-780 (#O323, eBiosciences), IgM-EF450 (#SA-DA4, eBiosciences), CD38-PerCP-Cy5.5 (#A60792, Beckman Coulter), CD93-PECy7 (#AA4.1, Biolegend). purified Rat anti-mouse CD16/CD32 (Mouse BD Fc Block™ BD Biosciences). For B cell receptor (BCR) stimulation, cells were cultured in 10 µg/mL AffiniPure F(ab')$_2$ fragment goat anti-mouse IgM, µ chain specific (Jackson Immuno Research). CD19-PE (#6D5, Biolegend), CD3-APCCy7 (#17A2, Biolegend), CD93-APC (#AA4.1, Invitrogen). Fc receptors blocked (Purified Rat Anti-Mouse CD16/CD32 (Mouse BD Fc Block™ BD Biosciences). Rabbit anti-TLR7 (D7; Cell Signaling Technology) and mouse anti mouseTLR7-PE (A94B10; BD Biosciences). CD19-BV650 (#HIB19, Biolegend), CD38-BV605 (#HIT2, Biolegend), CD24-BV711 (#ML5, BD Biosciences), IgD-BV510 (#IA6-2, Biolegend), CD27-APC-EF780 (#O323, eBioscience), CD11c-BUV395 (#B-ly6, BD Biosciences), CD3-BV786 (#SK7, BD Biosciences), CD56-BUV737 (#NCAM16.2, BD Biosciences), TLR7-PE (#4G6, Novus) and Myd88 (#EPR590(N), abcam).

Validation

All antibodies used were commercial antibodies and had been previously validated by the manufacturing companies. We further validated mouse TLR7-PE A94B10 antibody by staining splenocytes from Tlr7 knockout mice.
We provide the clones used for each antibody. Antibody titrations and dilutions used in each experiment are only relevant to the specific batch used, which change over time and therefore not useful.

Eukaryotic cell lines

Policy information about cell lines

Cell line source(s)	RAW267.7 cells were originally from the American Type Culture Collection (ATCC)
Authentication	The cell line used has not been authenticated by STR profiling
Mycoplasma contamination	All cell lines tested negative for mycoplasma contamination using PlasmoTestTM (InvivoGen).
Commonly misidentified lines (See ICLAC register)	The cell line used is not listed in the database of commonly misidentified cell lines

Animals and other organisms

Policy information about studies involving animals; ARRIVE guidelines recommended for reporting animal research

Laboratory animals

C57BL/6 Ncrl mice were used in this study. Both male and female mice were used and the sex has been identified in all figures by the genotype, adding the "Y" in all male genotypes, and specifying the allele of the X chromosomes: (+ or kik). Mice were used at 8-12 weeks for phenotyping and in vitro experiments, except organ histology which was examined at 26 weeks.

CFW/crl female mice from 6-16 weeks of age were used to mate with stud males for generation of CRISPR/Cas9 Tlr7 and Rnaseh2b gene edited mice.

Wild animals	The study did not involve wild animals.
Field-collected samples	The study did not involve field animals.
Ethics oversight	Animal experimentation was performed according to the regulations approved by the Australian National University's Animal Experimentation Ethics Committee.

Note that full information on the approval of the study protocol must also be provided in the manuscript.

Human research participants

Policy information about studies involving human research participants

Population characteristics	Individuals were either healthy controls, or patients who were diagnosed with systemic lupus erythematosus by treating physicians, or their family members. Individuals known medical treatments and clinical diagnosis are provided in Table S1.
Recruitment	Participants were recruited by their referring medical practitioners, based on clinical eligibility criteria, with the vast majority of eligible participants agreeing to participate in the study.<br>Young healthy controls for the phenotyping/RNAseq experiments of the proband with the TLR7 Y264H variant were recruited amongst young teenage girls within the same school in Canberra, of white European ascent (including one Spanish); thus gender, age, and ethnically matched to the proband (Spanish). However, other environmental / geographic influences (proband had been living in Guatemala for several years when last bled) could not be controlled for.
Ethics oversight	The study was approved by and complies with all relevant ethical regulations of the Australian National University and ACT Health Human Ethics Committees (2015/079), the University Hospitals Institutional Review Board, or by Renji Hospital Ethics Committee of Shanghai Jiaotong University School of Medicine.

Note that full information on the approval of the study protocol must also be provided in the manuscript.

Flow Cytometry

Plots

Confirm that:

☒ The axis labels state the marker and fluorochrome used (e.g. CD4-FITC).

☒ The axis scales are clearly visible. Include numbers along axes only for bottom left plot of group (a 'group' is an analysis of identical markers).

☒ All plots are contour plots with outliers or pseudocolor plots.

☒ A numerical value for number of cells or percentage (with statistics) is provided.

Methodology

Sample preparation	Human PBMCs were isolated using Ficoll-Paque gradient centrifugation and frozen thawed before staining for flow cytometric analysis. Single cell suspensions were prepared from mouse spleens and B cells were magnetically purified using mouse B Cell Isolation Kit (Miltenyi Biotec), labeled with Cell Trace Violet (CTV, Thermo Fisher) and cultured for 72 hours in complete RPMI 1640 media (Sigma-Aldrich) supplemented with 2mM L-Glutamine (GIBCO), 100 U penicillin-streptomycin (GIBCO), 0.1 mM nonessential amino acids (GIBCO), 100 mM HEPES (GIBCO), 55 mM β-mercaptoethanol (GIBCO) and 10% FBS (GIBCO) at 37°C in 5% CO2. Bone marrow was obtained from mice, the Fc receptors blocked and cells stained and sorted.
Instrument	Cells were sorted on a FACS Aria II, splenocytes and human PBMC samples were acquired on a Fortessa or Fortessa X-20 cytometer.
Software	FACS data was analyzed using FlowJo software v10 (FlowJo LLC).
Cell population abundance	Sorted sample purity was based on flow cytometry sorting analysis and stringent gating. Abundance of populations are indicated in the gating figures of the manuscript.
Gating strategy	SC-H/FSC-A (cells were gated along a diagonal gating strategy to eliminate cells with disproportional FSC-H and FSC-A size), SSC-W/SSC-H (cells with large SSC-W from scatter were eliminated), FSC-A/Live dead (cells staining negative for the live dead marker were selected as "live") and FSC-A/SSC-A (Cells were gated as lymphocytes if they had a lower size and granularity relative to other signals detected). Once cells were established as singlets, live and lymphocytes analysis was completed as described in the manuscript, where possible biphasic populations were used to identify positive and negative populations. The gating strategies used to identify individual subsets of interest have all been shown in the main figures.

☐ Tick this box to confirm that a figure exemplifying the gating strategy is provided in the Supplementary Information.
